# *In silico* characterisation of stand-alone response regulators of *Streptococcus pyogenes*

**DOI:** 10.1371/journal.pone.0240834

**Published:** 2020-10-19

**Authors:** Sean J. Buckley, Mark R. Davies, David J. McMillan

**Affiliations:** 1 School of Health and Sports Sciences, University of the Sunshine Coast, Sippy Downs, Queensland, Australia; 2 Department of Microbiology and Immunology, University of Melbourne at the Peter Doherty Institute for Infection and Immunity, Melbourne, Victoria, Australia; Ross University School of Medicine, DOMINICA

## Abstract

Bacterial “stand-alone” response regulators (RRs) are pivotal to the control of gene transcription in response to changing cytosolic and extracellular microenvironments during infection. The genome of group A *Streptococcus* (GAS) encodes more than 30 stand-alone RRs that orchestrate the expression of virulence factors involved in infecting multiple tissues, so causing an array of potentially lethal human diseases. Here, we analysed the molecular epidemiology and biological associations in the coding sequences (CDSs) and upstream intergenic regions (IGRs) of 35 stand-alone RRs from a collection of global GAS genomes. Of the 944 genomes analysed, 97% encoded 32 or more of the 35 tested RRs. The length of RR CDSs ranged from 297 to 1587 nucleotides with an average nucleotide diversity (π) of 0.012, while the IGRs ranged from 51 to 666 nucleotides with average π of 0.017. We present new evidence of recombination in multiple RRs including *mga*, leading to *mga-2* switching, *emm*-switching and *emm*-like gene chimerization, and the first instance of an isolate that encodes both *mga-1* and *mga-2*. Recombination was also evident in *rofA*/*nra* and *msmR* loci with 15 *emm*-types represented in multiple FCT (fibronectin-binding, collagen-binding, T-antigen)-types, including novel *emm*-type/FCT-type pairings. Strong associations were observed between concatenated RR allele types, and *emm-*type, MLST-type, core genome phylogroup, and country of sampling. No strong associations were observed between individual loci and disease outcome. We propose that 11 RRs may form part of future refinement of GAS typing systems that reflect core genome evolutionary associations. This subgenomic analysis revealed allelic traits that were informative to the biological function, GAS strain definition, and regional outbreak detection.

## Introduction

*Streptococcus pyogenes* (group A *Streptococcus*; GAS) colonises assorted human tissues causing multiple clinical manifestations, ranging from uncomplicated pharyngitis and impetigo to lethal invasive disease and post-infection sequelae [1]. GAS isolates are typically classified on the basis of nucleotide sequence variation in the 5’ end of *emm* gene, termed the *emm*-type [2]. Another typing scheme based on changes in the composition and arrangement of *emm* and *emm*-like genes, termed *emm*-pattern-type has been shown to be a reasonable correlate with tissue tropism: throat (type A-C), skin (type D), and throat or skin (type E ‘generalists’) [3].

The GAS genome encodes an arsenal of virulence factors and precise regulatory systems that confer adaptability in the face of challenging host environments [4]. Notwithstanding considerable recombination and genetic plasticity [5–11], the influence of GAS genotype diversity on differential clinical outcomes remains to be elucidated [1, 12, 13]. This is an important research focus, given that GAS kills more than 600,000 people globally each year [14].

GAS continuously sense the conditions in the surrounding environment whilst simultaneously regulating gene expression, allowing them to survive and thrive in the changing milieu throughout infection [4]. Unlike many other bacteria that employ multiple RNA polymerase sigma factors, GAS growth-phase gene expression is modulated globally by transcription response regulators (RRs) [15–18]. GAS RRs control factors that mediate metabolism, colonization of tissues, evasion of immunity, stressor response, dissemination, and persistence [19]. Whilst ‘two-component system’ RRs are encoded adjacent to a surface-exposed sensory kinase, ‘stand-alone’ RRs lack a hitherto-defined cognate sensory partner [19]. Stand-alone RRs possess helix-turn-helix domains that bind to DNA in the upstream intergenic region (IGR) of effector genes with a precision and affinity that varies with changes in intracellular conditions, such as the presence of an inducing substrate [20, 21]. Stand-alone RRs can interact with other RRs in complex transcription regulatory networks (TRNs), and are often auto-regulating [4, 20]. Although at least 30 virulence-related GAS stand-alone RRs are known, the full repertoire of stand-alone RRs remains to be characterised [4]. Consequently, the variability in the nucleotide sequences of the coding sequences (CDSs) [22] and IGRs of GAS stand-alone RRs is likely to contribute to differential biology and clinical outcomes. Hereafter RR refers to stand-alone RR unless otherwise stated.

Advances in whole-genome sequencing (WGS) and molecular characterisation have transformed the study of bacterial evolution, pathogenicity, and epidemiology [23]. WGS in many cases is faster, more cost effective, more auditable, and enables a higher resolution and discriminatory power than standard microbial methods [24–27]. Increasingly, it is evident that WGS will predominate as the bacteriological investigatory standard for typing, disease surveillance, disease manifestation, disease transmission, outbreak surveillance, evolution, vaccine development, and geotemporal variation [11, 26, 28–31]. However, the major impediments to the ascent of WGS are the development of standardised downstream bioinformatic analytical techniques [23, 26, 32], and high-quality, curated databases [33].

Here, we applied subgenomic analysis to GAS RRs, finding that whilst a range of sequence variation was observed in both the CDS and IGR sequences, with few exceptions they were present in all genomes. We observed that the same forms of mutation and recombination were present in both the CDSs and IGRs, suggesting a utility for IGRs in future genomic studies, and especially for RRs given that they are autoregulatory elements. Investigation of a specific recombination event in the IGRs of *mga* in a group of *emm*-pattern-type E generalist isolates led to the development of a putative evolutionary pathway for the deletion-fusion (chimerization) of genes within the *mga* regulon using multiple *emm*82 isolates in the dataset. We also ascertained that there was generally a higher degree of plasticity in many of the RR loci of the often clinically-relevant generalist isolates. Furthermore, we made multiple *emm*-type-specific observations that should inform *emm*-type selection of future wet assay and bioinformatics studies. One example of this was that the nucleotide sequences of both the *emm*3 *mga* CDSs as well as IGRs were different from their non-*emm*3 counterparts, suggesting a distinctive protein binding domain/DNA binding site pair. We also quantified many instances when an *emm*-type was represented in more than one multilocus sequence typing (MLST)-type and *vice versa*. We argue for augmentation of the current GAS typing schemes [11, 13], based on subgenomic interrogation of whole genome sequences. This study also reveals the utility of alternative schemes in cross-referencing, and defines the subgenomic resolution required for a functional GAS typing scheme.

## Methods

### Bacterial genomes and extraction of nucleotide sequence data

The 944 genomes tested in this study comprise 65 complete GAS genomes representing 27 *emm*-types sourced from the NCBI reference genomic database (as of 1st August 2018), and an additional 879 draft genomes representing 123 *emm*-types collected from five geographically disparate countries over the time period 1987 to 2013 (S1 Data) [11, 34–39]. A distribution of *emm*-types used in this study compared to the NCBI database of complete genomes and the Davies GAS atlas [11] is included (S1 Fig). Where available, the clinical data (for example, disease association, year of isolation, and country of isolation) was also collected for all genomes (S1 Data). Nucleotide sequences of the CDS and IGR of 35 selected stand-alone RRs were extracted from the genomes using the BLASTN algorithm implemented in Geneious 8.1.9 (maximum e-value of 1e-20) [40], and reconciled with annotated genes.

### Bioinformatic analyses

The nucleotide sequences of the CDS and IGR of the 35 RRs (S2 Fig and Table 1) were aligned using Muscle [41] as implemented in Geneious. Nucleotide polymorphisms were identified in both the CDSs and IGRs, and independently quantified using Geneious. Individual CDS and IGR alleles were subsequently defined on the basis of possessing a minimum of one Single Nucleotide Polymorphism (SNP) compared with all other alleles [12] (S2 and S3 Data, respectively).

**Table 1:** Distribution of GAS stand-alone response regulators.

RR^1^	Spy locus	Distribution (%)^2^	Function	References
*adcR*	spy0092	944/944 (100)	Zinc acquisition and virulence ^3^^,^^4^	[42]
*atoR*	spy1634	942/944 (99.8)	Short-chain fatty acid metabolism ^3^^,^^4^	[43]
*ccpA*	spy0514	944/944 (100)	Carbohydrate catabolite and virulence regulation ^3^^,^^4^^,^^5^	[44, 45]
*codY*	spy1777	944/944 (100)	Nutritional stress response ^3^^,^^4^^,^^5^	[46]
*comR*	spy0037	944/944 (100)	Biofilm-related transformation ^3^^,^^5^	[47]
*copY*	spy1717	944/944 (99.9)	Copper tolerance ^3^^,^^4^^,^^5^	[48]
*cpsY*	spy0898	944/944 (100)	Innate immunity defence ^3^	[49]
*crgR*	spy1870	944/944 (100)	Cathelicidin resistance ^3^	[50]
*ctsR*	spy2074	944/944 (100)	Heat stress response ^3^	[51]
*gczA*	spy0846	942/944 (99.8)	Zinc resistance efflux ^3^^,^^4^	[42]
*hrcA*	spy1763	943/944 (99.9)	Heat shock response repressor ^3^^,^^4^	[52]
*lrp*	spy1978	944/944 (100)	Not known, Under regulatory influence of *MsmR* in M49 background	[53, 54]
*malR*	spy1293	944/944 (100)	Cell adhesion and polysaccharide metabolism ^3^^,^^4^^, and^ ^5^ via saliva utilisation	[45]
*mga-1*	spy2019	151/944 (16)	Carbohydrate metabolite-responsive virulence regulator of ‘throat’ strains ^3^^,^^4^^,^^5^	[44, 55]
*mga-2*	spyM18_2077	794/944 (84)	Carbohydrate metabolite-responsive virulence regulator of ‘generalist’ strains ^3,4,5^	[44, 55]
*mrgA*	spy1259	942/944 (99.8)	ROS resistance via iron sequestration ^3^	[56]
*msmR*	spy49_0118	722/944 (75.5)	Regulation of type-dependent FCT and virulence genes ^3^	[54]
*mtsR*	spy0450	940/944 (99.6)	Metal uptake, virulence, and metabolism ^3^^,^^4^^,^^5^	[57, 58]
*nra*	spyM3_0097	328/944 (34.7)	Regulation of type-dependent FCT genes, *mga* regulon genes (via *Mga*), ERES genes (via *ralp3*), and other virulence genes. Primarily regulator of pilus genes (Danger thesis) ^3^ ^and^ ^4^ ^via *mga*,^ ^5^	[59, 60]
*perR*	spy0187	944/944 (100)	Metal homeostasis, oxidative environment response, and immunity defence ^3^^,^ ^4^ ^and likely^ ^5^	[61, 62]
*ralp3*	spy0735	834/944 (88.3)	Regulator of *epf* and *sagA* ^3^ ^and^ ^4^ ^via *mga*, and^ ^5^^.^	[63–65]
*regR*	spy0627	944/944 (100)	Expression of *hylA*, Under regulatory influence of RopB in NZ131 background	[66, 67]
*rgg2*	spy0496	940/944 (99.6)	Quorum sensing, biofilm regulator ^3^^,^^4^^,^^5^	[58]
*rgg3*	spy0533	940/944 (99.6)	Quorum sensing, biofilm regulator ^3^^,^^4^^,^^5^	[58, 68]
*rivR*	spy0216	944/944 (100)	*Mga* regulon genes (via *Mga*) ^3^ ^and^ ^4^ ^via *mga*,^ ^5^	[49, 69]
*rofA*	spy0124	616/944 (65.3)	Regulation of type-dependent FCT genes and toxins ^3^ ^and^ ^4^ ^via *mga*,^^5^	[20, 60]
*ropB*	spy2042	944/944 (100)	Growth phase-appropriate balance of virulence (e.g. *speB*) and metabolism ^3^^,^^4^^,^^5^	[68, 70]
*spy0715*	spy0715	944/944 (99.9)	GntR-like	[71]
*spy1202*	spy1202	944/944 (99.7)	GntR-like under regulatory influence of RopB in NZ131 background	[70]
*spy1258*	spy1258	944/944 (100)	TetR-like	[72]
*spy1602*	spy1602	942/944 (99.8)	GntR-like	
*spy2177*	spy2177	944/944 (99.8)	TetR-like under regulatory influence of RopB in NZ131 background.	[70]
*srv*	spy1857	940/944 (99.6)	*Mga* regulon genes (via *Mga*), CovRS regulated genes (via CovRS) and toxins ^3^^,^^5^	[73]
*vfr*	spy0887	944/944 (99.9)	Influences speB expression ^3^^,^^4^ ^via ropB, and^ ^5^	[74]
*vgl*	spy0188	676/944 (71.6)	Not known	[75]

^1^Gene name, or SF370 locus tag where not available

^2^Full length intact

^3^Virulence-related

^4^Metabolism-related

^5^Biofilm formation-related.

Nucleotide diversity (π) was calculated using DnaSP version 5.10.01 [76]. Allelic diversity was calculated using the *Simpsons Index of Diversity* [77] and the *Wallace coefficient* [78] as implemented at www.comparingpartitions.info. The ratio of non-synonymous (K_A_) to synonymous (K_S_) nucleotide substitution (K_A_/K_S_) was calculated in Mega7. Absent genes were designated as absent. The truncated proteins possess a premature stop codon that were almost all either caused by an indel that resulted in a frameshift mutation, or a non-synonymous point mutation. Phylogenetic relationships were inferred from the nucleotide sequences of individual RRs using the maximum likelihood algorithm, with a general time reversible model and bootstrap value of 1000 [79]. Analysis of both ‘recent’ and ‘ancestral’ recombination events, was performed using fastGEAR with default parameters. The resolution of an isolate type (defined by the concatenation of all of the RR CDS allele types for each strain) was tested for its ability to discriminate *emm*-type. The equivalent RR IGR type resolution was also tested. Alignment and visualization was performed using the BRIG tool [80].

Associations between the RR alleles and the typing, geotemporal, and clinical metadata, where tested using two methods. Firstly, neighbour-joining phylogenetic trees were constructed using the MEGAX maximum composite likelihood and uniform rates model (bootstrap = 1000) based on Muscle alignments of the 35 individual RR CDSs. This was also performed on the concatenated SNPs of the 35 RR CDSs (n = 3551) using a Muscle alignment of 3884 sites in length. Trees were labelled with metadata using Phandango. Secondly, concordance of the RR allele types and metadata was measured using the *Simpson Index of Diversity* and the adjusted *Wallace coefficient*.

## Results

### Distribution of GAS stand-alone response regulators

Overall, the CDSs of stand-alone RRs and their IGRs were well conserved throughout the 944 genomes examined. The majority of the genomes (n = 577) possessed DNA sequences that were highly homologous at all 35 RR loci (percentage nucleotide identity >95%). Only 2% of the genomes lacked four or more RRs (n = 20). The three genes that were most frequently absent were *vgl* (~28%), *msmR* (~24%), and *ralp3* (~12%) (Table 1, S2 and S3 Data).

### Diversity and variability in the coding sequences of selected GAS stand-alone response regulators

The size of RR CDSs ranged from 297 bp for *vfr* to 1587 bp for *mga-1* (Table 2). Nucleotide polymorphism causing allelic variation in the RR CDSs was primarily due to SNPs. The RR CDSs also exhibited single and multiple nucleotide indels. Single nucleotide indels were observed in *atoR*, *comR*, *copY*, *gczA*, *mrgA*, *rivR*, *spy0715*, *spy1258*, and *vfr*, while multi-nucleotide indels were observed in *atoR*, *comR*, *crgR*, *malR*, *mga-1*, *mga-2*, *spy0715*, and *spy1258*. The number of unique alleles per RR ranged from 25 for *ctsR* and *spy1202* to 201 for *mga-2* (Table 2). Based on *Simpson diversity index* (D) the ten most variable RRs alleles were *lrp*, *mtsR*, *rivR*, *atoR*, *spy1602*, *spy0715*, *regR*, *ccpA*, *rgg3*, and *copY* (Table 2). Multiple RRs including *atoR*, *mga-1*, *comR*, *copY*, *lrp*, *ralp3*, *regR*, *rivR*, *spy0715*, *spy1202*, *spy1258* and *spy1602* displayed variation in the nucleotide sequence and allelic length suggesting that some RRs can accommodate more sequence variation than others. How this relates to function, is unknown noting that variation in the function of GAS RofA response regulators has even been linked to SNPs [81]. Table 2 summarises the key measures of nucleotide diversity of the RR alleles. Together the 35 RR CDS loci could be used to identify 525 unique concatenated RR-types within the 944 genomes.

**Table 2:** Nucleotide variation in the coding sequences of selected GAS stand-alone response regulators.

Gene^1^	Size (nt)	Alleles	Variant nt positions^2^	Nucleotide diversity (π)	Allelic diversity (D)	Average nt percentage identity [range]	Recombination events^3^
*adcR*	723	32	41	0.0035	0.708	100 [94–100]	1
*atoR*	993	141	168	0.0173	0.981	98 [93–100]	3
*ccpA*	645	87	62	0.0037	0.962	100 [99–100]	0
*codY*	999	58	41	0.0041	0.915	100 [99–100]	1
*comR*	1203	78	407	0.0896	0.897	86 [52–100]	1
*copY*	894	87	182	0.0737	0.954	92 [65–100]	3
*cpsY*	432	63	58	0.0027	0.895	100 [99–100]	0
*crgR*	480	74	57	0.0041	0.933	100 [97–100]	1
*ctsR*	741	25	18	0.0025	0.616	100 [99–100]	0
*gczA*	459	61	55	0.0033	0.864	100 [97–100]	3
*hrcA*	639	95	89	0.0062	0.952	99 [97–100]	0
*lrp*	1038	164	202	0.0134	0.985	99 [90–100]	3
*malR*	717	90	187	0.0031	0.953	100 [95–100]	2
*mga-1*	1587	40	362	0.0181	0.278	98 [89–100]	3
*mga-2*	1491	201	366	0.0103	0.939	99 [84–100]	4
*mrgA*	1491	56	61	0.0026	0.838	100 [91–100]	0
*msmR*	780	90	83	0.0047	0.922	100 [99–100]	0
*mtsR*	465	145	87	0.0102	0.983	99 [98–100]	1
*nra*	885	40	50	0.0052	0.559	99 [99–100]	1
*perR*	741	44	35	0.0038	0.845	100 [99–100]	0
*ralp3*	1506	161	390	0.0065	0.926	97 [1–100]	0
*regR*	441	123	388	0.0292	0.967	97 [86–100]	4
*rgg2*	849	63	52	0.0027	0.917	100 [99–100]	0
*rgg3*	909	77	60	0.0053	0.958	99 [99–100]	1
*rivR*	840	156	140	0.0034	0.982	100 [99–100]	2
*rofA*	1533	122	201	0.0088	0.871	99 [96–100]	2
*ropB*	864	132	138	0.0036	0.945	100 [94–100]	1
*spy0715*	531	114	240	0.0453	0.971	95 [78–100]	17
*spy1202*	720	45	118	0.0041	0.571	100 [89–100]	1
*spy1258*	519	57	53	0.0019	0.703	100 [97–100]	0
*spy1602*	1032	135	232	0.0193	0.98	98 [90–100]	2
*spy2177*	540	54	100	0.0049	0.887	99 [65–100]	0
*srv*	903	52	37	0.0039	0.894	100 [99–100]	0
*vfr*	297	39	30	0.0011	0.602	100 [99–100]	0
*vgl*	846	32	36	0.0052	0.819	99 [95–100]	0

^1^Gene name, or SF370 locus tag where not available

^2^Variant nucleotides in the multiple sequence alignment

^3^Number of putative recombination events inferred by fastGEAR.

### Diversity and variability in the amino acid sequences of selected GAS stand-alone response regulators

Of the selected RRs, there were approximately twice as many repressors (or putative repressors) than activators. In a previous study we noted that all 14 of the GAS two-component system RRs possess helix-turn-helix (HTH) domains at their C-termini [82]. By contrast, 27 of the RRs tested had HTH domains at their N-termini, four were at the C-termini (*codY*, *lrp*, *msmR*, and *srv*), two were mid-protein (*adcR* and *ctsR*), and two lacked known HTH motifs (*vfr* and *vgl*). The majority of translated CDSs were intact and full length. However, there were many examples of significant variability in the composition and length of the translated proteins that suggested putative altered function (that is, loss or gain of function). The majority of truncations were observed in the C-terminal half of the translated sequence. In many cases the variants displayed *emm* gene association suggesting clonality. The number of nonsense mutations per RR ranged from 0 in CrgR, CtsR, and HrcA to 94 in AtoR (Table 3). Six RR proteins (CopY, GczA, RivR, Spy0715, ComR, and AtoR) had more than ten nonsense mutations. Observed causes of these truncations in the whole dataset included single nucleotide deletion (for example subset of *emm*1 *copY*), single nucleotide insertion (as in a subset of *emm*4 *ralp3*), and multiple nucleotide insertions (as in a subset of *emm*71 *comR*). The average amino acid (aa) identity values ranged from 77% for ComR to greater than 99% for 27 of the 35 RRs (Table 3). Collectively this implies that the most conserved of the proteins tested were HrcA, CtsR, CrgR, CpsY, Rgg3, and Srv, suggesting that evolution is constrained for some GAS RRs and not others.

**Table 3:** Amino acid variation in the coding sequences of selected GAS stand-alone response regulators.

Gene^1^	Average aa percentage identity [range]	Nonsense mutations^2^	π_A_/π_S_	K_A_/K_S_	Selection pressure^3^
*adcR*	99.3 [15.9–100]	3	0.069	0.068	neg
*atoR*	94.6 [9.6–100]	94	0.15	0.132	neg
*ccpA*	99.8 [16.6–100]	3	4.548	4.456	pos
*codY*	99.8 [34–100]	2	1.062	1.008	pos
*comR*	77.2 [7.5–100]	47	0.613	0.659	neg
*copY*	88.7 [19.4–100]	29	0.984	0.951	neg
*cpsY*	99.7 [77.5–100]	1	0.153	0.155	neg
*crgR*	99.5 [96–100]	0	0.185	0.107	neg
*ctsR*	99.4 [97.4–100]	0	0.955	0.947	neg
*gczA*	95.1 [3.3–100]	29	1.91	2.039	pos
*hrcA*	99.5 [98–100]	0	0.122	0.121	neg
*lrp*	98.7 [22.9–100]	1	0.131	0.13	neg
*malR*	99.2 [31.7–100]	15	0.573	0.455	neg
*mga-1*	93.4 [11.3–100]	5	0.109	0.108	neg
*mga-2*	98.5 [16.5–100]	9	0.785	0.909	neg
*mrgA*	99.4 [9–100]	4	0.027	0.025	neg
*msmR*	99.6 [88.4–100]	3	0.091	0.096	neg
*mtsR*	97.9 [6.7–100]	6	0.84	0.949	neg
*nra*	99.6 [98.4–100]	3	0.101	0.1	neg
*perR*	99.8 [98.1–100]	9	0.069	0.066	neg
*ralp3*	90.8 [5.5–100]	9	0.228	0.228	neg
*regR*	98.9 [9.5–100]	4	3.086	4.503	pos
*rgg2*	99.6 [97.9–100]	6	0.262	0.26	neg
*rgg3*	99.6 [78.4–100]	1	0.346	0.314	neg
*rivR*	96.2 [27.3–100]	36	0.413	0.548	neg
*rofA*	97.8 [15.7–100]	8	0.423	0.411	neg
*ropB*	99.3 [13–100]	15	0.098	0.099	neg
*spy0715*	94 [7.5–100]	60	0.689	0.723	neg
*spy1202*	99 [6.4–100]	13	0.062	0.052	neg
*spy1258*	98.2 [7.4–100]	19	0.272	0.311	neg
*spy1602*	98.1 [25.1–100]	2	0.204	0.203	neg
*spy2177*	97.8 [5.8–100]	11	0.788	0.554	neg
*srv*	99.9 [90.8–100]	1	0.018	0.018	neg
*vfr*	99 [10.2–100]	7	0.703	0.833	neg
*vgl*	98.9 [50–100]	3	0.507	0.506	neg

^1^Gene name, or SF370 locus tag where not available

^2^Alleles containing premature stop codon

^3^pos = positive and neg = negative.

### Diversity and variability in the upstream intergenic regions of selected GAS stand-alone response regulators

The RR IGRs ranged in size from 51 bp for *perR* to 666 bp for *mga-2* and 675 bp for *mga-1* (S2 Fig and Table 4). Again, most of the observed allelic variation in the RR IGRs was due to SNPs. However, there were also examples of single nucleotide indels, multiple nucleotide indels, and variable number of tandem repeats (VNTRs), and phage-related Insertion Sequences (IS). Single nucleotide indels were observed in the IGRs of *atoR*, *comR*, *copY*, *crgR*, *ctsR*, *hrcA*, *lrp*, *malR*, *mga-1*, *mga-2*, *msmR*, *nra*, *rgg3*, *rofA*, *spy0715*, *srv*, and *vgl*. While multi-nucleotide indels were observed in the IGRs of *atoR*, *ccpA*, *crgR*, *hrcA*, *lrp*, *mga-1*, *msmR*, *ralp3*, *rivR*, *rofA*, *ropB*, *and spy0715*. Examples of VNTR-related polymorphism were observed in *mga-1* IGRs of *emm*3, and *mga-2* IGRs of *emm*82 and *emm*87 isolates. The number of unique IGR alleles per RR ranged from 3 for *rgg2* to 133 for *mga-2* and *lrp* (Table 4). Based on *Simpson diversity index* (D) the ten most variable IGRs were upstream of *lrp*, *ralp3*, *atoR*, *mga-2*, *rivR*, *msmR*, *malR*, *comR*, *spy1602*, and *copY* (Table 4). Several of the intergenic loci, including *mga-2*, *atoR*, *copY*, *comR*, *lrp*, *ralp3*, *spy0715*, and *vgl* displayed variation in the allele length and, or nucleotide composition that was consistent with discrete allelic forms. Table 4 summarises the key measures of nucleotide diversity including allele-types, polymorphic nucleotide sites, nucleotide diversity, and *Simpson diversity index* (D) of the RR IGR alleles. Together the 35 RR IGR loci could be used to identify 473 unique concatenated RR-types within the 944 genomes.

**Table 4:** Nucleotide variation in the upstream intergenic regions of selected GAS stand-alone response regulators.

RR^1^	Size^2^	Alleles	Variant nt positions^3^	Nucleotide diversity (π)	Allelic diversity (D)	Recombination events^4^
*adcR*	109	12	12	0.0017	0.178	0
*atoR*	200	94	105	0.0002	0.966	0
*ccpA*	173	18	67	0.0066	0.515	0
*codY*	217	30	27	0.0072	0.801	0
*comR*	131	68	67	0.0148	0.879	0
*copY*	171	90	244	0.2906	0.814	0
*cpsY*	221	23	29	0.0036	0.610	0
*crgR*	239	27	32	0.0041	0.643	0
*ctsR*	195	15	15	0.0050	0.693	0
*gczA*	135	28	85	0.0074	0.756	0
*hrcA*	134	25	37	0.0052	0.580	1
*lrp*	360	133	214	0.0297	0.985	2
*malR*	245	106	213	0.0028	0.900	0
*mga-1*	675	42	396	0.0233	0.310	2
*mga-2*	666	133	535	0.0174	0.939	3
*mrgA*	162	31	71	0.0095	0.729	0
*msmR*	384	60	55	0.0143	0.904	1
*mtsR*	142	27	75	0.0019	0.321	0
*nra*	429	26	126	0.0173	0.564	0
*perR*	51	7	6	0.0014	0.149	0
*ralp3*	513	132	521	0.0308	0.970	1
*regR*	63	8	8	0.0126	0.515	0
*rgg2*	88	3	11	0.0006	0.147	0
*rgg3*	79	14	20	0.0125	0.777	0
*rivR*	554	80	109	0.0095	0.938	1
*rofA*	262	42	91	0.0021	0.759	1
*ropB*	268	44	182	0.0080	0.786	0
*spy0715*	147	34	190	0.0122	0.719	0
*spy1202*	55	7	5	0.0040	0.215	0
*spy1258*	117	20	19	0.0053	0.483	0
*spy1602*	105	41	40	0.0344	0.859	1
*spy2177*	134	34	66	0.0111	0.528	0
*srv*	105	6	13	0.0001	0.148	0
*vfr*	147	16	15	0.0008	0.150	0
*vgl*	155	27	63	0.0024	0.793	0

^1^Gene name, or SF370 locus tag where not available

^2^Nucleotide distance between RR genes and upstream gene

^3^Variant nucleotides in the multiple sequence alignment

^4^Number of putative recombination events inferred by fastGEAR.

Finally, short Open Reading Frames (ORF) of unknown function were identified upstream of *rofA*, *nra*, *ralp3*, *rivR*, *mga-1*, *mga-2* and *msmR* whose length and nucleotide identity were consistent with regulatory elements previously described upstream of *ropB*, *rgg2 and rgg3* [83, 84]. The size of the currently-annotated IGRs of these seven genes is larger than 100 bp, which is the average IGR length of GAS [85]. This suggests a putative biological function for these short ORFs, possibly as regulatory elements.

### Evidence for recombination in the stand-alone response regulator loci

Recombination was observed to span, flank, or intersect both the IGRs and CDSs of the RRs, and was at times caused by insertion sequences or VNTRs. The number of recombination events inferred for the RR CDSs using fastGEAR ranged from 17 for spy0715 to none for 14 of the genes (Table 2). While the equivalent range for the IGRs was zero for 26 of the genes and three for *mga-2* (Table 4). There was no significant difference between the mean number of recombination events inferred for the RRs and the GAS MLST loci [11]. The most recombinogenic intergenic alleles were *mga-2*, *mga-1*, and *lrp* with three, two, and two events inferred, respectively. Detailed descriptions of *mga*, *rofA*/*nra*, *msmR*, and FCT-types are provided in the sections below. The *mga* is of biological and clinical significance as it is known to influence expression of about 10% of the GAS genome and the transcription of *mga* is auto-regulating [4].

**Fig 1:**
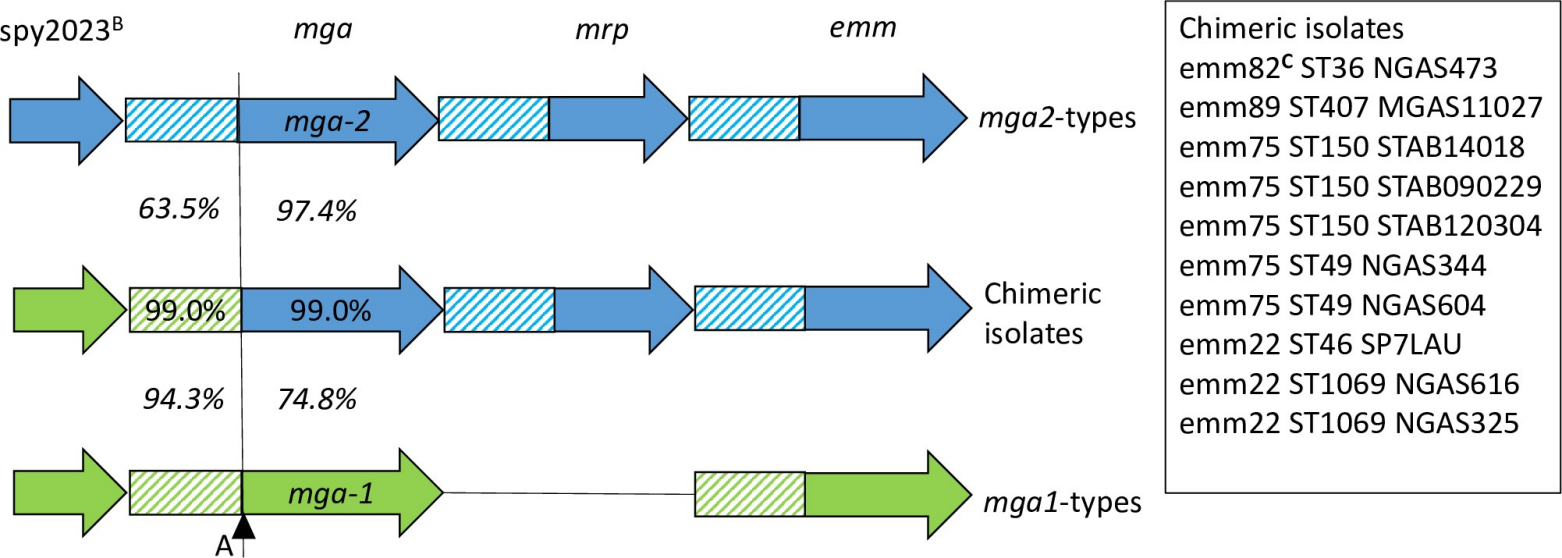
Schematic representation of the recombination event observed in the intergenic region of *mga* suggesting recombination of *mga-2* into an *mga-1*-type background (n = 10). Numerical values represent both the intra-allele and inter-allele percentage nucleotide identities. Hatched fill = Intergenic regions, Blue = genes of *mga-2*-type isolates, and Green = genes of *mga-1*-type isolates. Notes A = recombination flank, B = SF370 spy locus tag, C = *emm*82^C^ chimeric *emm* gene.

**Fig 2:**
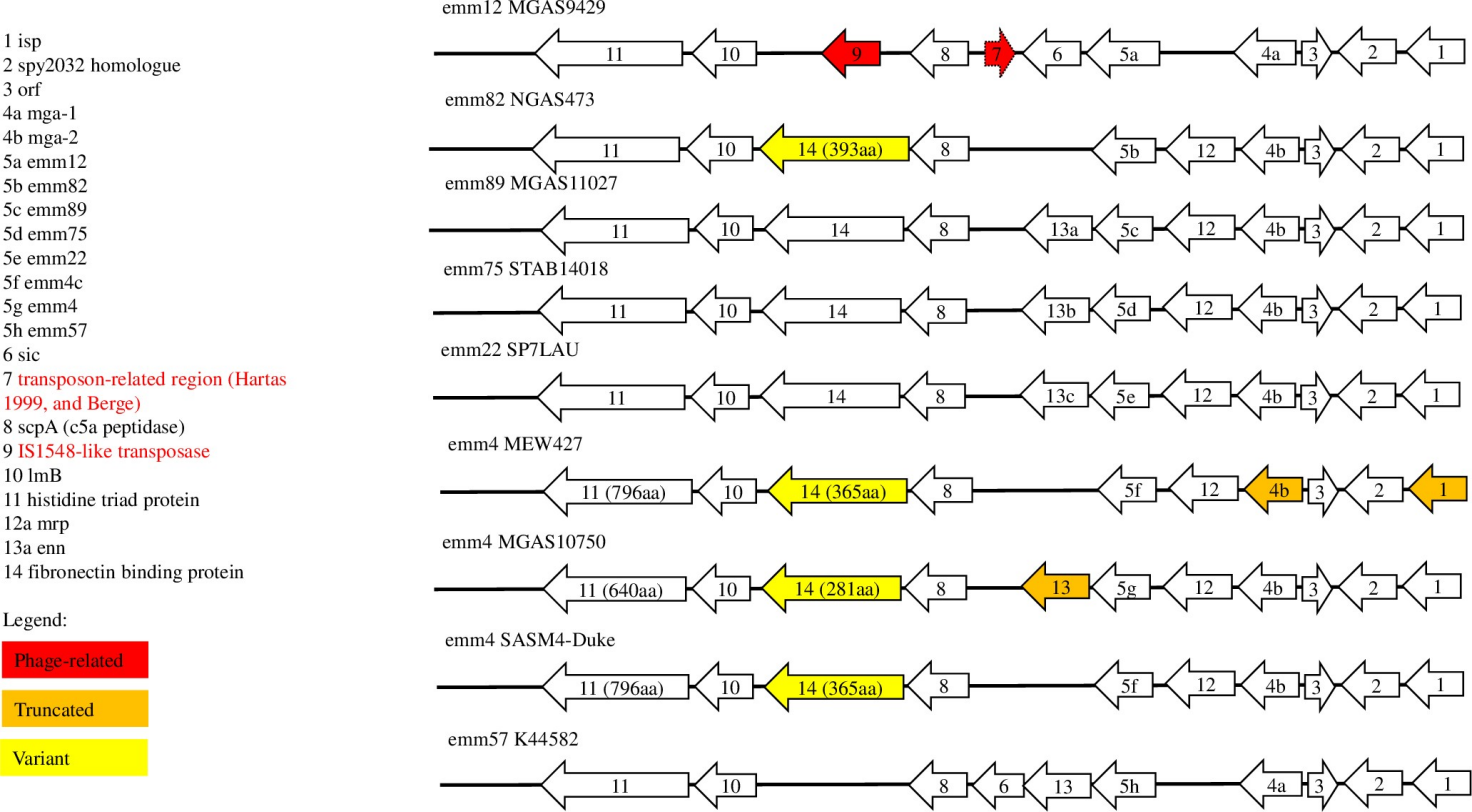
Schematic representation of the genes of the *mga* regulon depicting observed plasticity.

Sequence similarity and phylogenetic clustering of the combined CDS and IGR of *mga* in a subset of isolates (n = 10) strongly suggested that DNA encoding *mga-2* has homologously recombined into the flank of the intergenic locus of *mga-1* (Fig 1). That is, the *mga* IGRs and CDSs of these ten isolates displayed high pairwise nucleotide identity (99.0%), while sharing lower homologies with the IGRs (63.5%) and CDSs (74.8%) of *mga-2* and *mga-1* type isolates, respectively (Fig 1 and S3 Fig). These isolates were: NGAS473 ST36 (*emm*82), MGAS11027 ST407 (*emm*89), SP7LAU ST46 (*emm*22), NGAS325 ST1069 (*emm*22), NGAS616 ST1069 (*emm*22), STAB14018 ST150 (*emm*75), STAB120304 ST150 (*emm*75), STAB090229 ST150 (*emm*75), NGAS344 ST49 (*emm*75), and NGAS604 ST49 (*emm*75) (Fig 2). Fig 2 also depicts the general plasticity encompassing the *mga* regulon.

**Fig 3:**
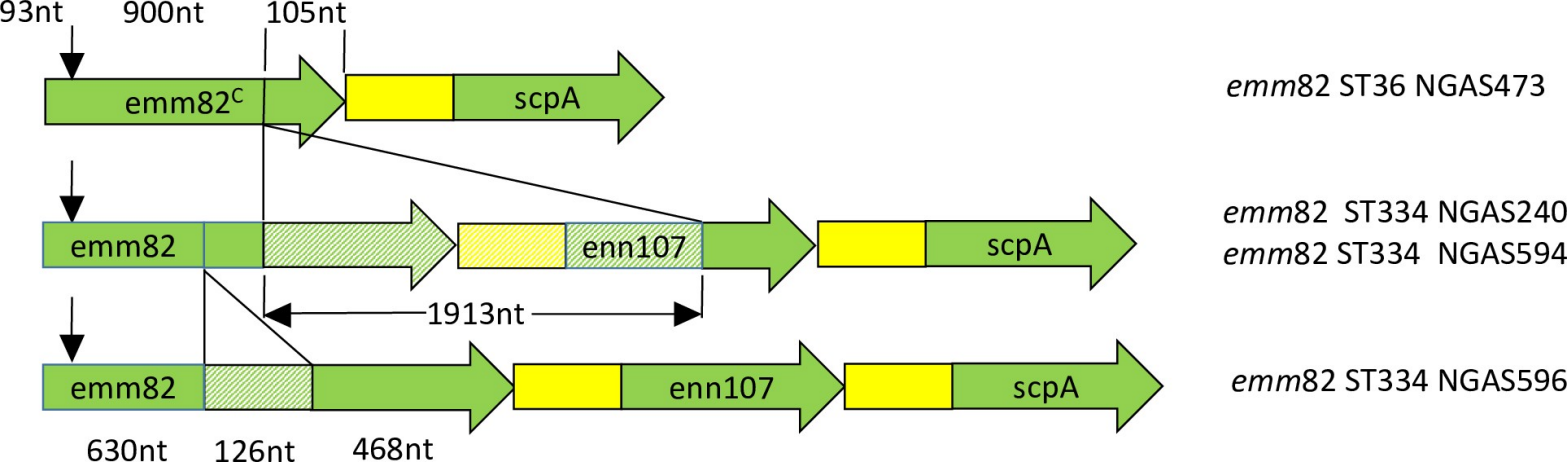
Proposed evolutionary pathway describing the deletion-fusion (chimerization) event observed in *emm*82^C^ NGAS473. The non-deleted DNA sequences share 100% percentage nucleotide identity. Green = Coding sequence, Yellow = Intergenic region, Hatched = Deleted DNA, and black arrow located 93 nt from the 5’ end depicts the start of the Centre for Disease Control 180 nt *emm*82.0 subtyping sequence.

While screening the IGRs of *mga* it was observed that one of these isolates, NGAS743, also displayed chimerization of *emm*82 and the adjacent *enn* gene, in contrast to other *emm*82 isolates in the dataset (Fig 3). Alignment of the *mga* regulon locus of the other *emm*82 isolates in the dataset revealed a putative evolutionary pathway to NGAS473 involving multiple deletion events (Fig 3). Multiple sequence alignment of the RR allele types groups NGAS473 with the *emm*12 isolates. Furthermore, *emm*82 NGAS473 is MLST-type ST36 which is historically observed in *emm*12 isolates. This association has recently been shown to be attributed to orthologous recombination of a region encompassing *emm*82 into an *emm*12 background [30]. Within this dataset ‘*mga-2* switching’ (n = 10) and ‘*emm*-switching’ was observed in isolates sampled from the United States of America, Canada, Lebanon, and France. Together these findings highlight the plasticity of the *mga* regulon in *emm*82 GAS, and identify an ‘*mga-*2-switching’ event in addition to an *emm*-switching in a GAS strain known to be clinically-relevant in northern hemisphere outbreaks [86].

Analysis of the *emm*-type and MLST-type pairings revealed numerous examples of *emm*-types that had multiple MLST-types, and MLST-types that were also found in multiple *emm*-types. Of the 256 unique MLST-types in the total dataset, eight (3.1%) were present in multiple *emm*-types and 17 (6.6%) were present in multiple *emm*-subtypes. Of the 125 unique *emm*-types, 67 (53.6%) had multiple MLST-types, while of the 186 unique *emm*-subtypes, 60 (32.3%) had multiple MLST-types. The five *emm*-types represented in the highest number of different MLST-type backgrounds were all *emm*-pattern-type ‘E’ (‘generalist’). While closely related clonal complexes and Single Locus Variants (SLVs) account for many of the occurrences of an *emm*-type occurring in multiple MLST-types, putative *emm*-switching is also contained in this subset. Collectively, these findings highlight both the shortcomings of using only *emm*-typing in strain definition, and the increased resolution that MLST can provide.

The FCT locus encodes the *rofA/nra* locus and *msmR* response regulators along with the key pili-associated, collagen-binding, and fibronectin-binding virulence genes, in an approximately *emm*-type-dependent manner [87]. Consistent with the observation of others, *rofA* and *nra* were mutually exclusive within each genome and were generally congruent with *emm*-type [87]. However, five genomes had an atypical *rofA/nra* to *emm* association (S4 Fig). These were Fijian isolates *emm*15.1 ST872 (20111V1I1) and *emm*18.22 ST535 (20058V1I1), and Kenyan isolates *emm*42.3 ST721 (K42600), *emm*49.9 ST705 (K36294), and *emm*57.0 ST723 (K44582). The scarcity of these MLST-types was evident because they were unique amongst their *emm*-type (*emm*18), and *emm*-subtypes for the other four. These five *emm*-subtypes were all either *emm*-pattern-type E or ‘single protein clade Y’. Interestingly, invasive GAS (iGAS) K44582 also encodes *sic* between *enn* and *scpA*, and lacks *crs* between *spy0778* and *rpsU*. This is to our knowledge the first recorded *emm*57 isolate with this gene configuration (Fig 2). There were no examples of an MLST-type that was represented in both *rofA*-positive and *nra*-positive isolates.

In this study all genomes from *emm*73 (n = 18) and *emm*105 (n = 10) isolates encoded *rofA*, whereas the two *emm*29 (n = 2) possessed *nra*. This contradicts others who observed *nra* in *emm*73 and *emm*105, and *rofA* in *emm*29 [59]. Furthermore, *emm*49-, *emm*68-, and *emm*110-type isolates were represented by both *msmR*-positive and *msmR*-negative genomes, and *emm*68 type isolates encoded one of three variants of *rofA*. While the above mentioned *emm*49.9 ST705 K36294 lacked *msmR*, it encoded an iron transporter in the FCT region displaying 100% identity with the equivalent *emm*77 gene (n = 2) and high sequence identity (80%) with a *Streptococcus canis* gene. Interestingly, within the dataset there were two *emm*-subtype 91.0 genomes isolated from canines that were of MLST-type 12, which is the MLST-type also represented in subtypes *emm*29.1, *emm*29.14, and *emm*29.2. This suggests recombination in the *mga* regulon and raises the possibility of inter-species recombination.

Collectively, this is further evidence of both *emm*-types encoding multiple FCT-types and the plasticity of the FCT locus. Based on the alignment of RR allele-types and *sof*, K36294 (*emm*49.9 ST705) appears to be a novel example of *emm*-switching, with a recipient genome of *emm*-type *emm*77. Isolate K44582 (*emm*57.0 ST 723) has also undergone rearrangement of the *mga* regulon locus, including putative *emm*-switch into an *emm*238 recipient with a fusion event at the 3’ end of the chimeric *emm*-like genes. GAS *emm*-types can display different FCT-types, albeit with low frequency. Within this dataset, *emm*-types encoding the unexpected *rofA*/*nra*-type were only observed in isolates sampled from Kenya and Fiji (n = 5). Qualifying these exceptions has implications for both isolate typing, and understanding the expression of pilus and biofilm formation.

### Associations between RR allelic profiles, and typing, geotemporal and clinical metadata

In order to assess relationships between the metadata and the nucleotide sequences of the RRs, phylogenetic and concordance analyses were performed. The phylogenetic analysis revealed no strong associations between the individual RR CDS alleles and *emm*-type, geotemporal data, or clinical outcomes. In general, the more discernible clustering was observed for metadata labels of the concatenated RR alleles phylogenetic tree (S5 Fig). Of note, were the ‘*emm*-pattern’ and ‘Country’ labels which displayed a greater degree of clustering. It was also noted that *ralp3* and *vgl* alleles were absent in the acute rheumatic fever (ARF)/rheumatic heart disease (RHD)-related isolates. Concordance between the concatenated RR allele types and various genomic traits (metadata) was tested, where the adjusted *Wallace coefficients* values represent the mean likelihood that two identical concatenated RR allele types share the same metadata value (S4 Data). The concatenated RR alleles were highly predictive of the *emm*-type and *emm*-subtype and by inference *emm*-cluster and *emm*-pattern. Adjusted *Wallace coefficients* between concatenated RR allele type and *emm*-type, MLST, and core genome phylogroup were measured as 99.8%, 98.3%, and 99.7%, respectively. Each of the concatenated RR allele types was observed in isolates of only one *emm*-type in all except for two cases. That is, where one type was seen in both *emm*101 (n = 3 of 11) and *emm*205 (n = 1 of 1), and another in both *emm*183 (n = 1 of 7) and *emm*79 (n = 1 of 3). However, 100 of the 125 *emm*-types had more than one concatenated RR allele type. Suggesting that the concatenated allele type is more predictive of the *emm*-type than *vice versa*. Similarly, the concatenated RR alleles were highly predictive of the country of sampling (91.5%). Moreover, when the *emm*-subtype and country of sampling were amalgamated, the adjusted *Wallace coefficient* increased to 93.4%, suggesting a geographical dependency in the variability of the RR alleles. Finally, within this dataset, the chance of two concatenated RR allele types sharing the same site of tissue sampled and disease outcome were, 58.4% and 63.3%, respectively, suggesting that the RR alleles have less power in predicting GAS clinical outcomes than they do for the evolutionary history of a strain. However, it should be noted that the *emm*-types of the isolates that have switched *mga-2* have been previously implicated in antibiotic resistance by others [88–91].

### Evidence for selection pressure on response regulators

Values for the ratios π_A_/π_S_ and K_A_/K_S_ were calculated for each of the RR coding alleles (Table 3). These values generally correlated and suggested that the majority were under negative selection pressure. Several exceptions, inferring positive pressure were observed for *ccpA*, *codY*, *gczA*, and *regR*.

### *rofA*-like protein (RALP) genes and *msmR*

It has previously been established that *rofA*, *nra*, *ralp3* and *rivR* are the *rofA*-like proteins (RALPs), and together with *msmR* are significant regulators of the virulence-related FCT and ‘*eno ralp3 epf sagA’* (ERES) loci [20, 54]. They share approximately 62% aa identity, and all GAS isolates encode either *rofA* or *nra*, but not both [59]. Very few *emm*-types are represented in multiple FCT-types, and more specifically the *rofA*/*nra*-type of an isolate correlates tightly with *emm*-type [92]. *rofA* and *nra* are auto-regulating, global virulence regulators that generally exert positive and negative influence on the FCT regulon, respectively, in an FCT-type-dependent manner [4]. Linkages have also been observed between *emm*-type and the form and function of *ralp3* and *msmR* [20, 54]. The RALPs contain N-terminal helix-turn-helix (HTH) DNA-binding domains and mid protein or C-terminal phosphotransferase system regulating domains (PRDs).

The throat-associated MGAS10750 is an *emm4* GAS reference genome that lacks *hasA*, encoding hyaluronan synthase, a key enzyme involved in synthesising the hyaluronic acid capsule a key determinant of the pathogenicity of GAS [93]. Recently, a chimeric fusion of *emm4* and the adjacent *enn* gene was characterised, designated *emm4c*, noted for its current clinical importance, and identified in the Paediatric Autoimmune Neuropsychiatric Disorders Associated with *Streptococci* (PANDAS)-associated throat isolate MEW427 [94, 95]. Other *emm4c*-encoding isolates have been associated with invasive GAS outbreak and non-synonymous variation of *ropB* and increased *speB* transcription [96].

In this study, all genomes were observed to encode either *rofA* or *nra*; *emm*-types encoding multiple FCT-types are detailed above (n = 5). *ralp3* was present in 834 of the 944 genomes, representing 114 *emm*-types of which 10 (*emm* 18, 19, 22, 53, 68, 75, 80, 83, 89, and 111) were also represented in isolates that lacked *ralp3*. In line with previous studies, we identified *ralp3* in *emm*-types 1, 4, 12, 28, and 49 [97], and can also report the first instance of a naturally occurring *ralp3* in an *emm53* isolate (n = 2 of 6: ST460 from Kenya and ST363~ from Fiji) [98]. Each of the NCBI ARF-associated genomes (*emm*5 Manfredo, *emm*6 JRS4, *emm*6 JRS4_DNA, *emm*6 D471, *emm*14 HSC5, *emm*18 MGAS8232, and *emm*23 M23ND) that were ‘single protein *emm*-cluster clade Y’ representatives lacked *ralp3*. Of the *emm*89 isolates tested (n = 33), *ralp3* was only present in the *emm*-subtypes 89.14 (n = 9) and 89.8 (n = 4). Only two of the six *emm*53 encoded *ralp3*, and these alleles were different. The *mga* allele of these two *emm*53 isolates were different from each other, and the other four *emm*53 *mga* alleles (including reference strain AP53). The *msmR* gene was present in 722 of the 944 genomes representing 101 *emm*-types of which 12 (*emm* 8, 12, 19, 22, 25, 49, 68, 77, 82, 92, 110, and 238) were also represented in isolates that lacked *msmR*. *msmR* was not encoded in any *emm*4 genomes. We observed a shorter *ralp3* variant in *emm*12 (S4 Fig), which has traditionally been considered amongst the most ‘nephritogenic’ strains [99].

Truncation of Nra by a stop codon has been described previously in *emm*18 MGAS8232 [100]. We also only observed the truncation of Nra in *emm*18 (n = 3 of 15 including MGAS8232). Truncation of RofA was observed in a single representative of seven different *emm*-types including the NCBI genomes pharyngeal *emm*6 MGAS10394, invasive *emm*44 STAB901, and invasive *emm*59 MGAS15252. Variants of RivR were observed in *emm*3 (n = 12 of 12). Within the IGRs of *msmR*, multi-nucleotide insertions were observed in isolates representing *emm*89 (n = 19, including *emm*89 clades 2 and 3), *emm*1 (n = 1), *emm*9 (n = 1), and *emm*77 (n = 1). While, multiple putative CovR DNA-binding nucleotide sequence (‘ATTARA’) were observed in the IGRs of *nra*.

In our dataset, seven *emm4c*-encoding isolates were identified, and observed to possess a truncation of *ralp3* (n = 7 of 18) resulting in a protein 318 shorter than *ralp3* in MGAS10750. The nucleotide sequence variants of *emm4 ralp3* also correlated with the geographical location of sampling. Nonsense mutations were seen in *mga-2* and *isp* of MEW427, and *enn* of MGAS10750, while their fibronectin binding proteins were 365 and 281 long, respectively. Variants of *rivR* were observed in *emm*4 (n = 11 of 19 including MGAS10750 and MEW427) that were 88 aa and 191 aa long, respectively (compared with the 502 aa of M1GAS). All of the e*mm*4 genomes lacked *msmR*. Given that the *mga* regulon and FCT region influence virulence, and *ralp3* plays a role in GAS survival in blood [64], the variability of these genes suggests that they may play a regulatory role in *emm4c* virulence.

### Multiple gene regulator of GAS (*mga*)

GAS Mga is a metabolite-responsive, auto-regulating, global regulator of virulence genes encoded by two divergent alleles, *mga-1* and *mga-2*, that correlate with *emm*–type [92]. Each allele is respectively linked to a throat-associated serum opacity factor (*sof*)-negative phenotype, or a skin-associated or ‘generalist’ *sof*-positive phenotype [92]. This suggests an important role for *mga-1* and *mga-2* in the evolutionary history of GAS tissue tropism [92]. *mga* encodes two PRDs, between N-terminal HTH DNA-binding domains and a C-terminal EBII-like domain [101]. Mga indirectly affects expression of over 10% of the GAS genome particularly in the exponential growth stage [44, 102].

**Fig 4:**
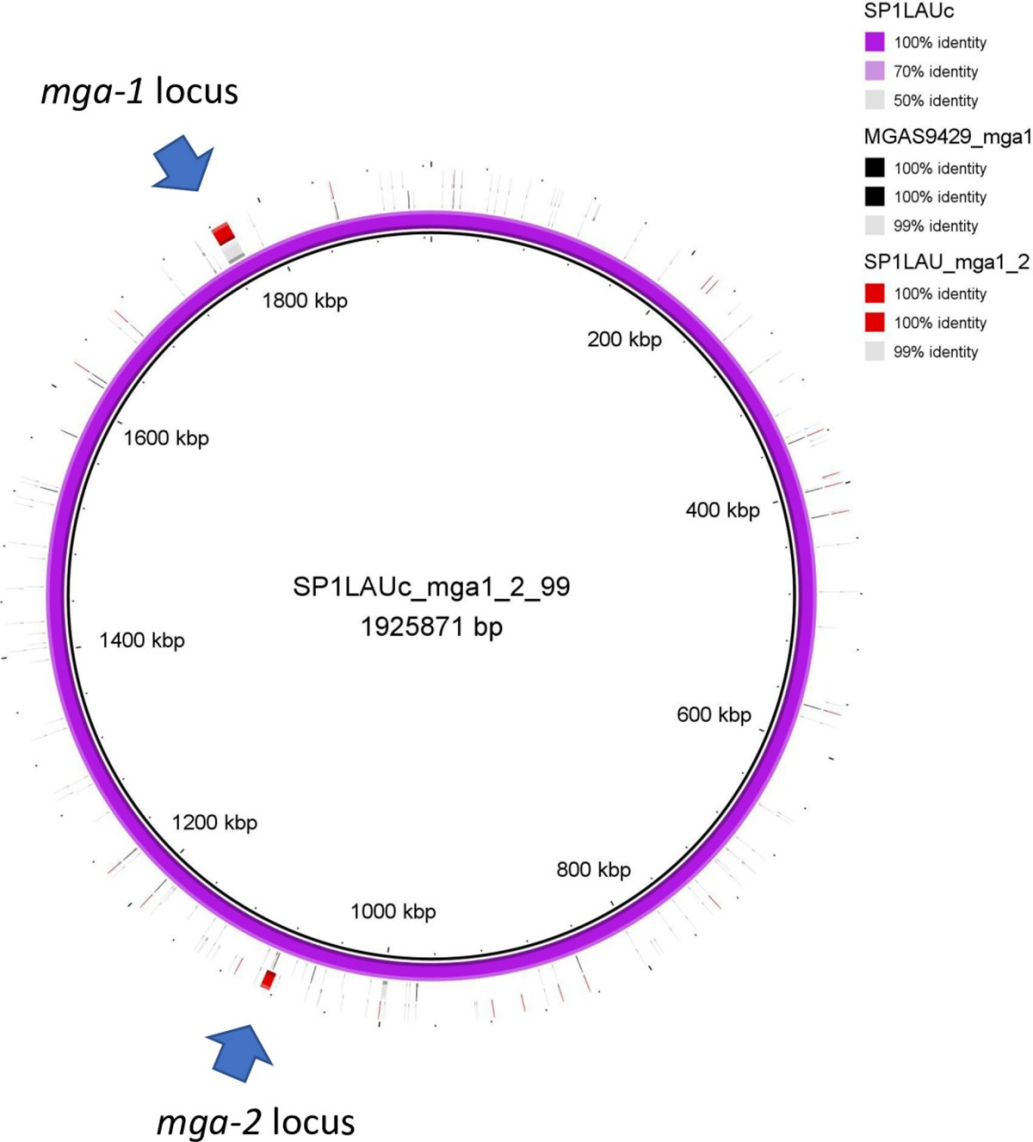
Subgenomic comparison of the *mga* loci of *emm*12 GAS isolates, SP1LAU and *emm*12 reference strain MGAS9429, displaying the presence of both *mga-1* and *mga-2* loci in SP1LAU using Blast Ring Generator (BRIG).

In this study *mga* was observed in all genomes, and the variants *mga-1* and *mga-2* displayed average intra-variant percentage identities of 97.7% and 98.8%, respectively. In a novel finding, throat-associated *emm*12 ST36 isolate (SP1LAU) encoded both *mga-1* and *mga-2* (Fig 4). In addition to an intact *rofA*, *ralp3*, and *rivR*, SP1LAU also encoded *mga-1* in the canonical locus, of the allele-type *mga*379 [103]. The *mga-2*-like gene displayed 91.1% similarity to the nucleotide sequence of *mga-2*, and was encoded 8963 nucleotides downstream of the phage-related DNase (*spd1*) between recombinase (*recT*) and a gene encoding a phage subunit. This finding is of significance in the understanding of gene expression in clade I *emm*12 GAS [104].

**Fig 5:**
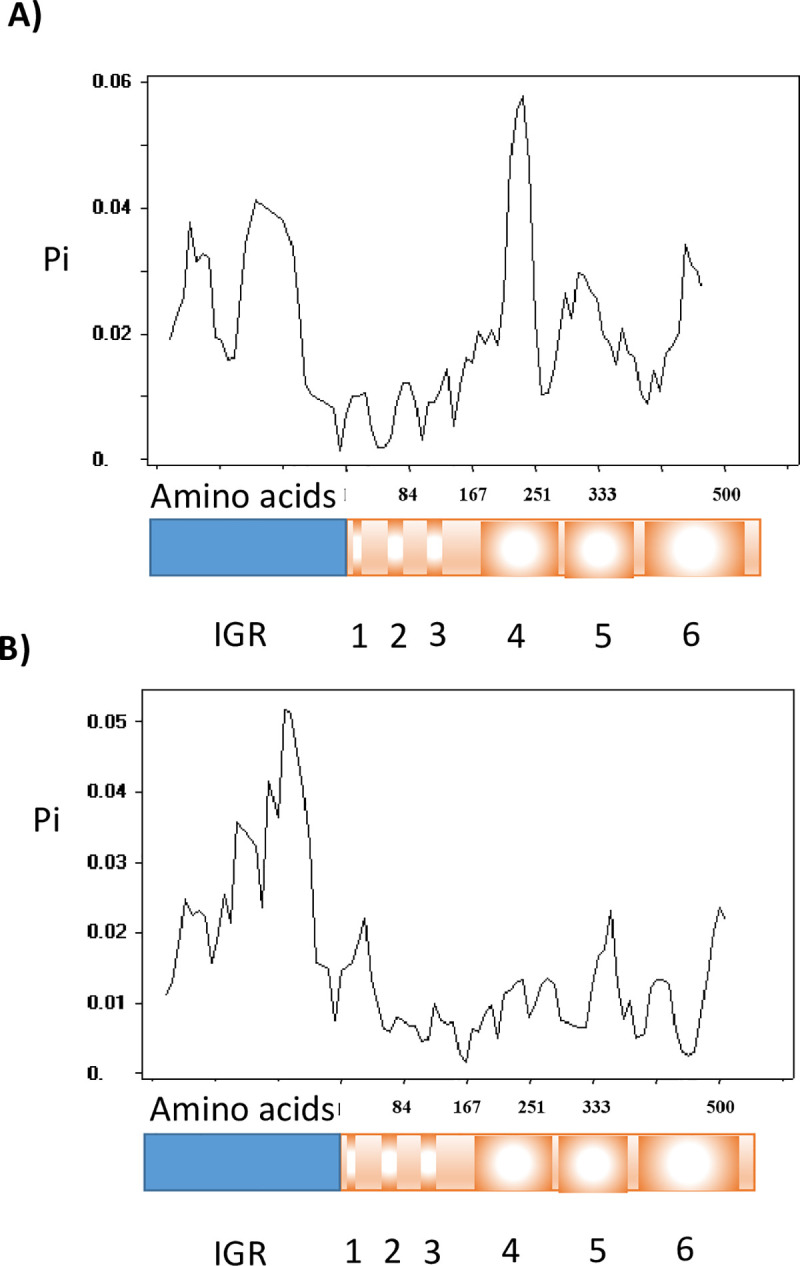
Observed nucleotide diversity (π) distribution within the intergenic region and functional domains of A) *mga-1* (n = 151) and B) *mga-2* (n = 793). IGR = intergenic region, 1 = Common Mga domain, 2 = helix-turn-helix (HTH)-3, 3 = HTH-4, 4 = phosphotransferase system regulatory domain (PRD)-1, 5 = PRD-2, 6 = phosphotransferase system enzyme IIB-like domain.

DNA polymorphism in the IGRs and CDSs of *mga-1* and *mga-2* was calculated and plotted using DnaSP sliding window algorithm (Fig 5). The IGR of *mga-2* displayed a higher degree of polymorphism than the equivalent of *mga-1*, with the greatest difference observed at its 3’ end adjacent to the coding region. While the coding region of *mga-1* had greater polymorphism than *mga-2*, displaying bands of peak variation that were consistent with the previously described functional domains. The domain displaying the greatest variability was the PRD-1 domain. These findings inform the relative variability of the functional domains of the *mga* [44, 105], and are consistent with the recombination event described above.

Considering *emm*3 GAS, serotype-specific mutations of *rocA*, *fasC*, and *rivR* have been observed [106, 107]. Flores et al. have described a VNTR in the IGR of *emm*3 *mga-1* whose variable number of repeat units (two or three) correlated with the asymptomatic carrier and invasive phenotypes, respectively [108]. Within our dataset the three repeat unit variant of the VNTR was observed exclusively in the *emm*3 *mga-1* IGRs (n = 12 of 12). Additionally, an 18 nt insertion (886nt-904nt) in the PRD-2 domain was also seen exclusively in *emm*3 *mga-1* coding region (n = 12 of 12). Our findings raise the possibility that the relationship between the distinctive CDSs and IGRs of *emm*3 *mga* may influence the binding specificity of *emm*3 *mga* in regulating its own transcription. We present these data as another example of *emm*3-specific variability, and as a putative marker for *emm3* GAS [109].

Further variation in *mga* is summarised as follows. Sanson et al. identified a non-synonymous H201R mutation, which significantly increased virulence of clinically relevant *emm*59 [110]. We identified the same mutation in *emm*59 MGAS1882 (*emm*-cluster-type E6) and seven other genomes, representing *emm*73 (E4), *emm*94 (E6), *emm*102 (E4), and *emm*114 (E4). We noted that *emm*5 Mga-1 (n = 2 of 2 including Manfredo) possessed a truncation of the C-terminus that yields a translated sequence that is 37 aa shorter than that of *emm*1 M1GAS. Mga-1 was truncated by nonsense mutations in *emm*1 MTB313, and four *emm*12 isolates, while Mga-2 was truncated in *emm*44 STAB901, *emm*4 MEW427 (Fig 2) and *emm*80 Rosenbach. Collectively, these findings are further evidence of the plasticity of *mga* in cluster E-type GAS.

### ropB-like proteins: *ropB*, *rgg2*, *rgg3*, *and comR*

*ropB*, *rgg2*, *rgg3*, and *comR* are the *rgg*-like genes that are present in all GAS strains [58]. RopB is the growth phase-dependent, global regulator that controls the expression of multiple virulence genes including *speB* during high cell density [111]. Vfr acts as an inhibitory peptide in the RopB-dependent expression of SpeB [111]. Rgg2 and Rgg3 bind post-translationally to short hydrophobic peptides (SHPs), which are encoded proximally, in an inter-related fashion to regulate the transcription of a common set quorum sensing-related genes [58]. Similarly, ComR interacts with the SigX-inducing peptide (XIP) to upregulate transcription of competence genes [112, 113] and is essential in *emm*3-type biofilm formation [47]. Different *comR* allele variants have been identified in *emm*3 MGAS315 and *emm*1 MGAS5005 [47]. The functional domains of these ComR-types have been investigated and found to show different biological activity [114]. The *mga*, RALP, and *ropB*-like genes contrive complex and yet to be elucidated transcriptional regulatory networks that have proven growth phase- and serotype-dependencies [4, 20].

In this study, *comR* was present in all genomes tested. Phylogenetic analysis revealed the novel finding that each of the 944 genomes tested encoded one of the two distinct allele types (S4 Fig). ComR-1 (represented by *emm*3 MGAS315) and ComR-2 (represented by *emm*1 MGAS5005), displayed 99.1% and 99.8% intra-type identity at the protein level, respectively, and 58.1% between types. *comR*-1 and *comR*-2 were represented in 78 and 79 *emm*-types, respectively. Thirty two *emm*-types (4, 15, 18, 19, 22, 25, 28, 42, 49, 53, 57, 60, 63, 65, 68, 70, 75, 77, 82, 83, 84, 89, 90, 93, 110, 116, 118, 122, 169, 192, 209, and 223) where represented in both *comR* types. The above mentioned *emm*49.9 ST705 K36294 was the only *emm*49 isolate (n = 1 of 9), and the Kenyan-sampled *emm*89.8 isolates were the only *emm*89 isolates (n = 4 of 33) to encode comR-1. The most variable ComR-2-related *emm*-types were *emm*11, *emm*25, *emm*49, *emm*71, *emm*82, *emm*106. While for ComR-1 the most variable were *emm*25 and *emm*83. A subset of *emm*25 ComR-1 (n = 21) displayed a 15 aa variant at the C-terminus due to a frameshift caused by a single adenine deletion. Multiple sequence alignment of the ComR-2 set revealed ten variant proteins that had a three aa insertion from 201–203 aa (‘ELD’ in NZ131; and ‘EQF’ in one ST591 *emm*82.1, one *emm*106, and seven *emm*49 isolates). These loci coincide with a putative pheromone ligand-binding domain [114]. The variable *comR emm*-types, *emm*15, *emm*49, and *emm*82, are mentioned in the evidence of recombination section above. While *emm*82 and *emm*49 (NZ131) display increased competence [115], and *emm*25 and *emm*49 are poststreptococcal glomerulonephritis-associated *emm*-types [116]. Therein, the described variation in the functional domains of *comR* are likely to inform the biology of competence and biofilm formation, and the clinical importance of the included *emm*-types.

The *rgg*-like genes were well conserved reinforcing the importance of their roles in the fitness of GAS. Several noteworthy examples of variation include the following. In MEW427, RopB displayed T104I and S116L mutations (with respect to MGAS10750), and Vfr was truncated, again suggesting different regulatory mechanisms between the *emm*4 isolates. A 145 nt sequence that contains multiple putative CovR DNA binding sites has inserted into the IGRs of *rgg2*, and *ropB* in five genomes (including *emm*89 ST407 MGAS11027 and four *emm*65.5 ST215 isolates). This suggests differential transcriptional regulation. While *emm*89 MGAS11027 has an indel in the IGR of *rgg3* that is unique amongst *emm*89 isolates. Both *emm*65 and *emm*89 have shown variability in biofilm production [117, 118]. While, a putative CovR DNA-binding sequences, was also observed upstream of *rgg2*. Our results advance the testable hypothesis, that measured intra-strain variability in the ability produce biofilm may provide insights into biofilm formation mechanisms.

### Other stand-alone response regulators (*crgR*, *lrp*, *copY*)

*crgR* is a transcriptional regulator that is important for survival in the presence of the antimicrobial peptide LL-37 in *emm*49 NZ131 [119]. Subsequent work with *emm*1 MGAS5005 and *emm*6 JRS4 has identified an *emm*-type-dependent biological activity [50, 120, 121].

In this study, *crgR* was encoded in all genomes, that when translated produced two variant protein lengths. The *emm*1 MGAS5005 was 5 aa shorter than the 253 aa *emm*6 JRS4 variant. The CovR DNA binding site was observed in a subset of the *crgR* IGRs, including *emm*6 but excluding *emm*1 isolates (S4 Fig). Therein possibly explaining differential expression of *comR*. Variation in the CDSs and IGRs of *crgR* may correlate with observed differential bioactivity (for example, variable functional efficacy in ll-37 resistance of the *crgR* of the two strains). Phylogenetic trees of the CDSs of *lrp* and *copY* have been included (S4 Fig) to illustrate the diversity within these loci.

### Isolates displaying wide-spread disruption to stand-alone response regulators

Several of the individual isolates showed a higher degree of variability across all of their RRs. These were *emm*1 MTB313, *emm*4 MEW472, *emm*44 STAB901, *emm*49 NZ131, *emm*82 and *emm*87 isolates. MTB313 is a ‘highly mucoid’ isolate that displays variability or truncation of AdcR, GczA, MalR, Mga-1, MrgA, MtsR, RegR, LacR and Spy2177. In addition to the genes described above, MEW472 variants of Ralp3, RopB and Vfr were also observed. *emm*44 STAB901 displayed variant AdcR, GczA, Mga-2, MtsR, RegR, RivR, RofA, LacR and Srv. While *emm*82 and *emm*87 are emerging clinically-relevant strains in North America [34].

## Discussion

### Distribution and diversity in the nucleotide and amino acid sequences of the IGR and CDS of GAS RRs

Here we characterised the distribution and diversity of the nucleotide and amino acid sequences of the IGRs and CDSs of 35 selected GAS RRs from 944 geotemporally diverse genomes. Different and often novel forms of variability were observed in the IGR and CDS loci, including single and multiple nucleotide mutations, recombination, and VNTRs. These individual nucleotide differences were used to define IGR and CDS allele types which then facilitated comparison to other existing typing schemes and inference of loci recombinogenicity and selection pressure. Because GAS RRs have been observed to be autoregulating [4, 20, 71], it was important to not exclude the IGRs from this study. Consequently, within the IGRs we identified several novel recombination events and putative binding sites including that of the global regulator CovR. We were also able to identify many instances of nonsense mutations, causing premature stop codons in the translated sequences that possibly alter the function of the protein by deleting key functional domains. We also provide new insight into the evolutionary dynamics of RR and IGR which clearly shows that carriage of these networks are important to GAS biology. Furthermore, the expansion of population genomic frameworks to capture RRs is required to get a better understanding of the nexus between regulatory systems, virulence pathways and pathogenesis.

### Recombination

The recombinogenicity of GAS is well established [5, 11]. By applying comparative subgenomic techniques we were able to increase the understanding of recombinogenicity of the RR loci, and identified several key loci involved in recombination events, including the *mga* regulon and FCT region. Specifically, we identified novel recombination events in the *mga* locus in ten isolates that were consistent with a switch from *mga-1* to *mga-2* type (“*mga-2* switching”). In one of these isolates, *emm*82 ST36 NGAS5949, we also observed a chimerization (deletion fusion) of the *emm*-like genes, for which we have proposed an evolutionary path using other *emm*82 isolates in the dataset. The discovery of *mga-2* switching is significant, because autoregulating *mga* is known to control the expression of about 10% of the GAS genome including surface-exposed M-protein and other virulence factors. *mga-1* is found in throat-associated GAS and is considered a proxy for *emm*-pattern-type A-C tissue tropism [92]. Therefore, recombination of *mga-2* into an otherwise *mga-1* genomic background is predicted to dramatically alter the transcription profile with the possible consequence of altering host-pathogen interaction in growth-phase transition. Our findings increase the understanding of *emm*82 GAS. Interestingly there has been a recent increase in *emm*82 outbreaks in North America, and in this context, investigation of the impact the *mga-2* switch on tissue tropism and virulence warrants further investigation.

We also identified the first known instance of an isolate (SP1LAU *emm*12 ST-type 36) encoding both an intact *mga-1* and *mga-2*, with the latter also having proximal bacteriophage-related elements suggesting a phage-mediated mode of recombination. Further investigation is required to assess the impact of this recombination event on the transcriptional landscape of SP1LAU.

GAS encodes either *rofA* or *nra* at the same locus in the FCT region. We observed five *emm*-types that were represented in both the *rofA*-positive and *nra*-positive subsets, that is five *emm*-types with multiple FCT-types. This was either explained by an *emm*-switch or recombination of the *rofA/nra* locus (that is, an FCT-switch), and resulted in several novel *emm*/FCT pairings. Identifying these pairings is useful in mitigating *emm*-type/FCT-typing ambiguities. This is of clinical significance, since *rofA* and *nra* are generally positive and negative regulators, respectively, of surface-exposed pili which are central to GAS biofilm formation. We also observed that each of the isolates of the *emm*-types represented in multiple FCT-types were sampled in Kenya or Fiji (n = 5), while the isolates displaying *mga-2* switching were sampled from northern hemisphere countries of higher median income. This raises the possibility of differential virulence factors associated with disproportionate rates of poverty, insecure and low-paid labour, poorer conditions and overcrowded housing. Regardless, a higher degree of overall plasticity was noted in *emm*-pattern E type generalist isolates, especially in the *mga* regulon and FCT region. Collectively, these findings could explain mechanisms for the geographically-dependent, rapid evolution of adhesion and immunity evasion in the progression of GAS disease.

### Selection pressure

One of the two response regulators inferred to be under the largest positive selection pressure was *regR* which represses expression of chromosomally-encoded hyaluronidase (*hylA*) [66]. While the mechanism has not been elucidated, *hylA* has been implicated in the degradation of both GAS and human hyaluronic acid, possibly enhancing the dissemination of GAS [66]. Historically the expression of an abundance of ‘mucoid’ GAS hyaluronic acid capsule, mediated by the *hasABC* operon, has been associated with virulent isolates. More recently virulent acapsular isolates, lacking intact and functional *hasABC*, have been observed [122]. We note that whilst acapsular isolates have been a topic of recent GAS virulence studies, the role of *hylA* and its regulator, *regR*, also warrant closer scrutiny.

### Associations

Strong associations were observed between the concatenated RR allele types and the current GAS typing systems. Weaker yet highly predictive associations were observed between concatenated RR alleles and country of sampling, and this was augmented with the amalgamation of the country of sampling with the isolate *emm*-type. Therein, suggesting a geographical dependence on the evolutionary history of GAS RR alleles. The power of the concatenated RR allele types to predict the clinical outcomes was significantly lower than for the typing and geotemporal metadata. In general, individual RR alleles were considerably less predictive of the metadata, however several notable observations were made. Switching of *mga-2* was observed in *emm*-types 22, 75, 82, and 89, all of which are *emm*-pattern-E types that have been associated with clinically-relevant antibiotic resistance [88–91]. Furthermore, SP7LAU was one of the isolates displaying *mga-2* switching as were isolates of *emm*22 ST46-type, which has been identified as one of the most frequently observed macrolide-resistant lineages [90]. Collectively, this serves to increase our understanding of the evolutionary history of GAS.

### Typing

The most commonly used epidemiological marker for GAS is the *emm*-type, and this is commonly used as a proxy for inferring evolutionary relatedness, especially within geotemporally restricted settings. However, in response to an immunity-imposed selection pressure, *emm* is known to readily mutate or recombine into a diversity of GAS genomic backgrounds [13, 123]. Another key GAS molecular typing scheme is the MLST which is based on the sequence of seven (partial) housekeeping genes. In combination the *emm*-type and MLST-type yield a less ambiguously defined GAS strain than *emm*-type alone. However, recombination has also been observed within the seven MLST housekeeping genes [13]. Amidst this complexity, measurement of the associations between *emm*-type and MLST-type has identified weakness in their definition of the GAS strain, particularly in isolates of *emm*-pattern types D and E from high-income countries [11, 124–126]. Comparison of *emm*-type and or MLST-type to the traditional serological surface-exposed GAS typing proteins (for example, M-protein, pili T antigens, and serum opacity factor) has also yielded some inconsistencies between these typing schemes [127, 128]. The complexity of GAS genomics adds to the difficulty of deciphering GAS biology and epidemiology, and has led to calls for reconsideration of the functional definition of a GAS strain [11, 13].

In this study we observed that the frequency with which an *emm*-type is not represented by a single MLST-type, and vice versa, was not *de minimus*. Using the RR allele types as a cross reference, we identified novel examples of *mga-2* switching and FCT-type/*emm*-type pairs, and inferred novel examples of the horizontal gene transfer of *emm* in a distantly related MLST-type (*emm*-switching). Together these observations serve to mitigate GAS typing ambiguities, and in the latter case add to the growing list of inferred *emm*-switches [13, 30, 125, 129]. Several of the observed advantages of typing using GAS RR alleles included the following. GAS RRs are a family of cytosolic proteins that share broadly similar functional domains and functions, including control of the expression of traditional GAS typing proteins. Nearly all RRs are found in all GAS genomes at loci that are distributed throughout the GAS genome. Targeting multiple RRs in a typing scheme reduces the reliance on a single locus, and the effect of recombination on typing schemes. We observed no difference in the mean recombination rates between the RR and MLST-typing loci. However, with judicious selection of RR alleles there were 11 that were inferred to be core and non-recombinogenic genes (Tables 1 and 2). Furthermore, RR alleles are contrived from the subgenomic interrogation of the nucleotide sequences of GAS WGSs which are available in increasing abundance and cost-effectiveness. RR alleles are genotype-dependent and not phenotype-dependent like traditional serological GAS typing proteins, circumventing some of the complexities of host-pathogen interaction. The proteins used in traditional typing schemes are generally antigenic and implicated in immune evasion, and as therein display high intragenic variability, experience strong selection pressure, and are prone to recombination. By contrast the RRs display a range of intragenic variability and recombinogenicity, and only two are inferred to be under strong selection pressure. In more nuanced observations it is preferred that a GAS typing scheme is readily backwards compatible with the abundant *emm*-type-specific knowledge base and is readily expandable to ensure future-proofing.

These findings are significant because they support the redefinition of a GAS strain by quantifying and mitigating elements of the existing typing ambiguities. They also re-iterate the notable plasticity of the *mga* regulon and FCT region of the *emm*-pattern-type E isolates, and therein identify a potential mechanism for the rapid evolution of E type isolates. They also serve to better inform the choice of *emm*-type in future GAS bioinformatic and laboratory studies. The delineation and description of genomic diversity may also indicate differential evolutionary history or virulence, with the associated downstream consequences for understanding GAS epidemiology and disease outcomes.

### Resolving power of RR allele-types

The future of microbiological molecular typing schemes will be WGS-centric. As such, choosing all 35 RR loci (as opposed to a selection of less than 35) may not provide significant impost. However, we acknowledge that MLST system is based on seven loci and any PCR-based RR typing system of equivalent discriminatory power would likely require a maximum of seven loci to justify adoption. At this stage the 11 RR alleles inferred to be core and non-recombinogenic are worthy of consideration. However, defining the minimum set of RR alleles that provides adequate power to discriminate a globally evolving population is the focus of future work.

Furthermore, the *mga* locus represents a noteworthy example of the importance of data resolution and granularity in subgenomics. Traditionally, GAS *mga* has been classified in two similar allele types *mga-1* and *mga-2*. Today, it seems logical and cost-effective to utilise the resolution of next-generation sequencing to define additional *mga* allele-types based on individual nucleotide variation [103]. However, moving forward it is important to realise the ongoing utility of the *mga-1* and *mga-2* variants, given their strong association with niche preference and tissue tropism. Furthermore, the work of others has identified that *emm*3 encodes distinctive naturally-occurring *mga-3* IGR alleles that are causal of differential virulence [108]. We observed that the IGR of *emm*3 *mga* was also distinctive in the Mga binding site, potentially representing an influential feature of autoregulation. Analogously, we observed that two previously described different *comR* alleles were present across the extent of our large dataset. Given that *comR* has been implicated in natural transformation and biofilm formation, definition of *comR-1* and *comR-2* promises equally distinct biological associations. In light of these examples, our findings increase the definition of the compartmentalisation and resolving power of the variation of IGR and CDS RR allele-types in deciphering bioinformatic, biological and clinical manifestations.

## Conclusions

We observed strong associations between the collective variation in the DNA sequences of the RR alleles, and GAS *emm*-type, MLST-type, core genome phylogroup, and the country of sampling. Our subgenomic interrogation of GAS genomes confirms the resolution and utility of RR loci in the burgeoning redefinition of GAS typing and strain. Whilst we saw no strong novel associations between individual RR loci and clinical outcomes, our work is likely to inform the selection of *emm*-type in future bioinformatic and laboratory studies. Furthermore, response regulators are clearly essential to the long term persistence of GAS, and a better understanding of how response regulators evolve/ relate to transcriptional networks is essential to deciphering the GAS host-pathogen interface.

## Supporting information

S1 Data Catalogue of GAS strains. Catalogue of NCBI and draft GAS genomes and metadata.
(XLSX)

S2 Data Typing data and concordance of response regulator CDSs. Typing data and concordance of response regulator CDSs.
(XLSX)

S3 Data Typing data and concordance of response regulator IGRs. Typing data and concordance of response regulator IGRs.
(XLSX)

S4 Data Concordance of metadata. Concordance analysis of genomic traits and metadata.
(XLSX)

S1 Fig *emm*-type distribution. Distribution of *emm*-types within this study (n = 125), the NCBI database of complete genomes as at 11-3-2020 (n = 59), and the Davies GAS atlas (n = 149) [11].
(PPTX)

S2 Fig Response regulator gene drawings. Schematic drawings of GAS response regulator genes.
(PPTX)

S3 Fig Dendrogram and phylogram of the *mga* CDSs and IGRs. Maximum likelihood and Neighbour-joining phylogenetic trees of the DNA sequences of *mga* CDSs and IGRs displaying recombination event, and the two *mga* alleles of SP1LAU.
(PDF)

S4 Fig Dendrograms of GAS response regulator CDSs and IGRs. Dendrograms of GAS response regulator CDSs (*comR*, *rofA*, *ralp3*, *lrp*, and *copY*) and IGR (*crgR*).
(PDF)

S5 Fig Phylogram of concatenated RR alleles. Neighbour joining phylogenetic tree of 3551 SNPs generated from an alignment of 35 response regulator genes.
(PDF)
