# Supplementary material for: In silico characterisation of stand-alone response regulators of Streptococcus pyogenes
Source: PLoS One. 2020 Oct 19;15(10):e0240834. doi: 10.1371/journal.pone.0240834 (PMC7571705; doi:10.1371/journal.pone.0240834)
Supplement: S1 Fig — Distribution of emm-types within this study (n = 125), the NCBI database of complete genomes as at 11-3-2020 (n = 59), and the Davies GAS atlas (n = 149) [11]. (PPTX) [file pone.0240834.s005.pptx]

## Slide 1
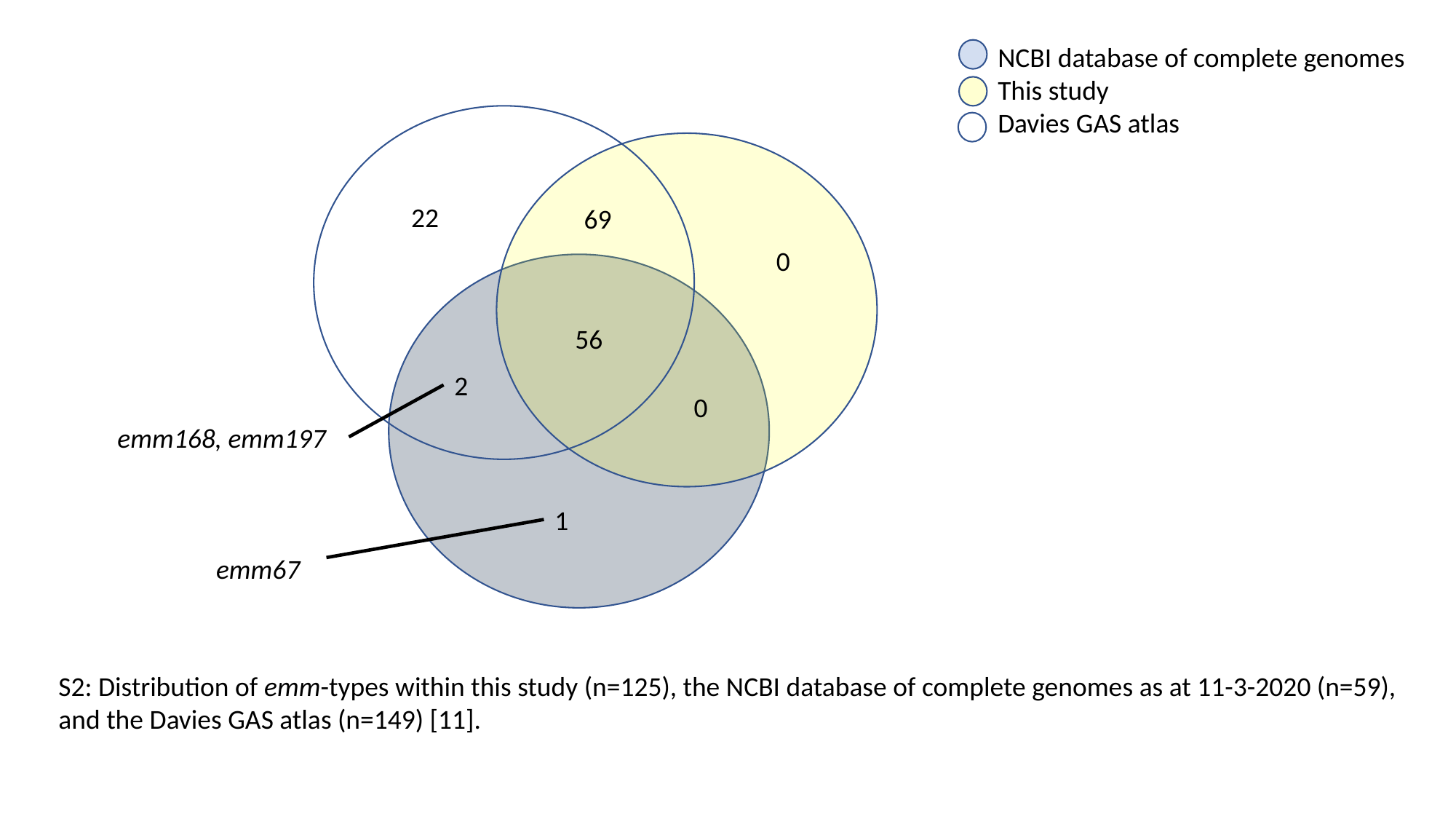

NCBI database of complete genomes
This study
Davies GAS atlas
22
69
0
56
2
0
1
emm168, emm197
emm67
S2: Distribution of emm-types within this study (n=125), the NCBI database of complete genomes as at 11-3-2020 (n=59), and the Davies GAS atlas (n=149) [11].
