# Supplementary material for: In silico characterisation of stand-alone response regulators of Streptococcus pyogenes
Source: PLoS One. 2020 Oct 19;15(10):e0240834. doi: 10.1371/journal.pone.0240834 (PMC7571705; doi:10.1371/journal.pone.0240834)
Supplement: S2 Fig — Schematic drawings of GAS response regulator genes. (PPTX) [file pone.0240834.s006.pptx]

## Slide 1
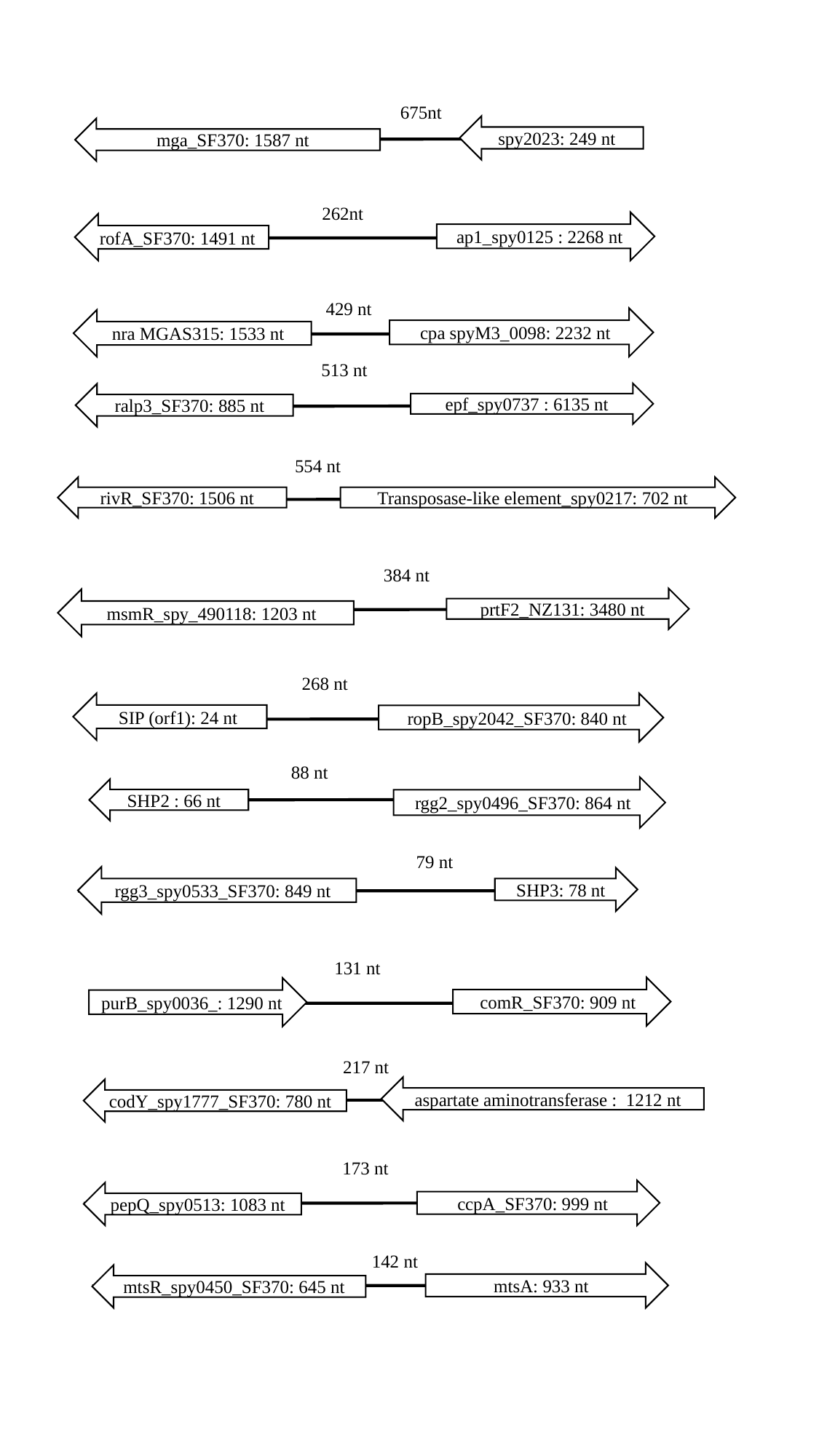

675nt
spy2023: 249 nt
mga_SF370: 1587 nt
 262nt
ap1_spy0125 : 2268 nt
rofA_SF370: 1491 nt
429 nt
cpa spyM3_0098: 2232 nt
nra MGAS315: 1533 nt
513 nt
epf_spy0737 : 6135 nt
ralp3_SF370: 885 nt
 554 nt
Transposase-like element_spy0217: 702 nt
rivR_SF370: 1506 nt
 384 nt
prtF2_NZ131: 3480 nt
msmR_spy_490118: 1203 nt
 268 nt
 SIP (orf1): 24 nt
 ropB_spy2042_SF370: 840 nt
88 nt
rgg2_spy0496_SF370: 864 nt
SHP2 : 66 nt
 79 nt
rgg3_spy0533_SF370: 849 nt
SHP3: 78 nt
131 nt
 comR_SF370: 909 nt
purB_spy0036_: 1290 nt
217 nt
aspartate aminotransferase : 1212 nt
codY_spy1777_SF370: 780 nt
 173 nt
ccpA_SF370: 999 nt
pepQ_spy0513: 1083 nt
 142 nt
mtsA: 933 nt
mtsR_spy0450_SF370: 645 nt

## Slide 2
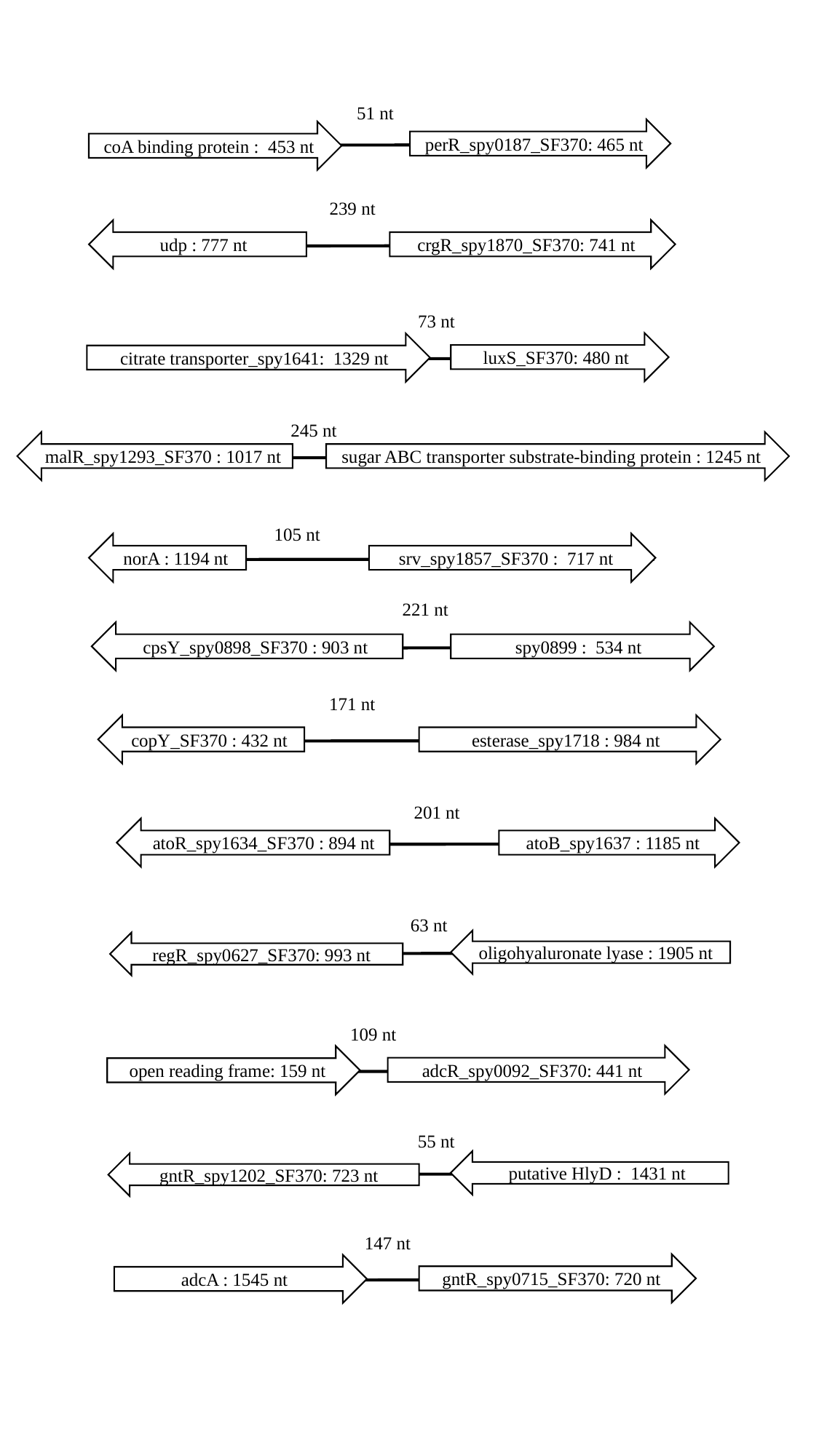

51 nt
perR_spy0187_SF370: 465 nt
coA binding protein : 453 nt
 239 nt
udp : 777 nt
crgR_spy1870_SF370: 741 nt
 73 nt
 luxS_SF370: 480 nt
 citrate transporter_spy1641: 1329 nt
 245 nt
 malR_spy1293_SF370 : 1017 nt
sugar ABC transporter substrate-binding protein : 1245 nt
 105 nt
srv_spy1857_SF370 : 717 nt
 norA : 1194 nt
 221 nt
 cpsY_spy0898_SF370 : 903 nt
 spy0899 : 534 nt
 171 nt
 copY_SF370 : 432 nt
 esterase_spy1718 : 984 nt
 201 nt
 atoR_spy1634_SF370 : 894 nt
atoB_spy1637 : 1185 nt
 63 nt
oligohyaluronate lyase : 1905 nt
regR_spy0627_SF370: 993 nt
109 nt
adcR_spy0092_SF370: 441 nt
open reading frame: 159 nt
 55 nt
 putative HlyD : 1431 nt
gntR_spy1202_SF370: 723 nt
147 nt
gntR_spy0715_SF370: 720 nt
adcA : 1545 nt

## Slide 3
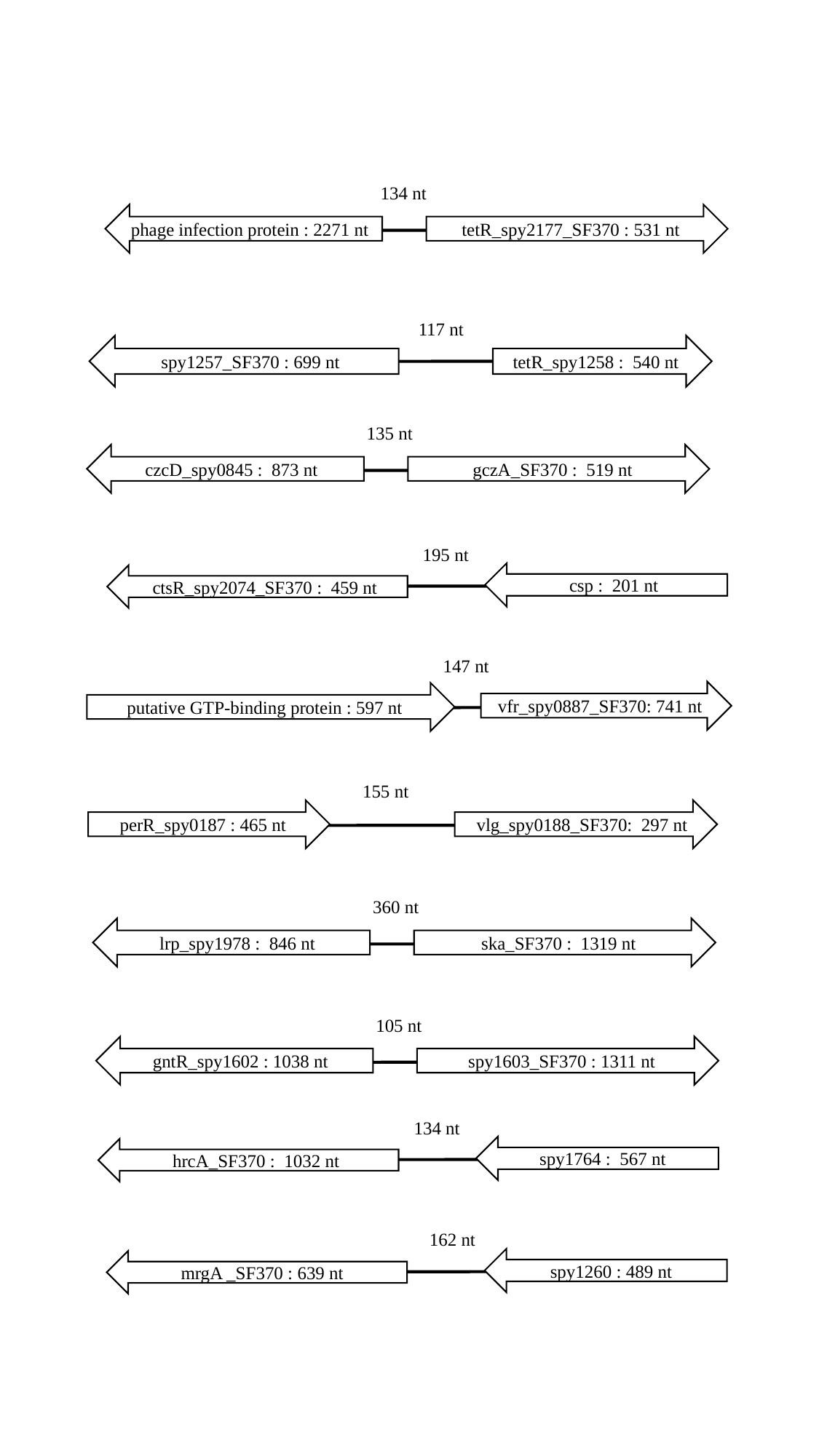

134 nt
phage infection protein : 2271 nt
tetR_spy2177_SF370 : 531 nt
 117 nt
spy1257_SF370 : 699 nt
tetR_spy1258 : 540 nt
 135 nt
czcD_spy0845 : 873 nt
gczA_SF370 : 519 nt
 195 nt
 csp : 201 nt
 ctsR_spy2074_SF370 : 459 nt
147 nt
vfr_spy0887_SF370: 741 nt
putative GTP-binding protein : 597 nt
 155 nt
perR_spy0187 : 465 nt
 vlg_spy0188_SF370: 297 nt
 360 nt
lrp_spy1978 : 846 nt
ska_SF370 : 1319 nt
 105 nt
gntR_spy1602 : 1038 nt
spy1603_SF370 : 1311 nt
 134 nt
spy1764 : 567 nt
 hrcA_SF370 : 1032 nt
162 nt
spy1260 : 489 nt
mrgA _SF370 : 639 nt
