# Supplementary material for: In silico characterisation of stand-alone response regulators of Streptococcus pyogenes
Source: PLoS One. 2020 Oct 19;15(10):e0240834. doi: 10.1371/journal.pone.0240834 (PMC7571705; doi:10.1371/journal.pone.0240834)
Supplement: S3 Fig — Maximum likelihood and Neighbour-joining phylogenetic trees of the DNA sequences of mga CDSs and IGRs displaying recombination event, and the two mga alleles of SP1LAU. (PDF) [file pone.0240834.s007.pdf]

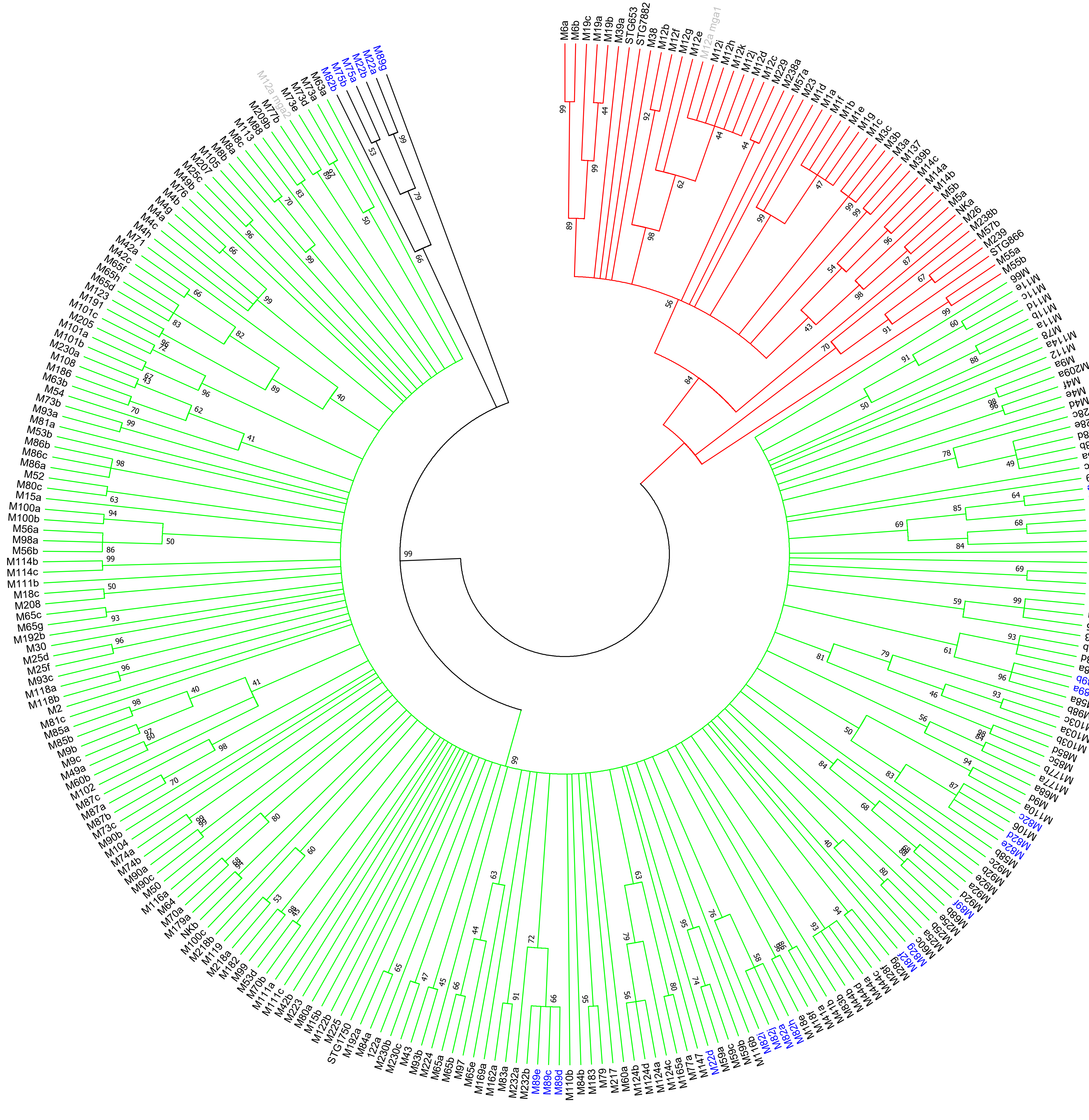

S6a: Dendrogram of the *mga-1* and *mga-2* intergenic and coding sequences in 944 genomes. The unique alleles in each of the 125 *emm*-types segregated into three clades corresponding to *mga-1* (red), *mga-2* (green), and the chimeric combination of *mga-2* downstream of a recombined *mga-1* intergenic region (black). Blue labels indicate the *emm*-types that segregate separately from the chimeric alleles, suggesting recombination at the chimerised loci. Silver labels represent the two *mga* loci observed in the *emm12* SP1LAU genome. Bootstrap values (percentage from 1000 replicates) of greater than 40% are shown at the bifurcating nodes.

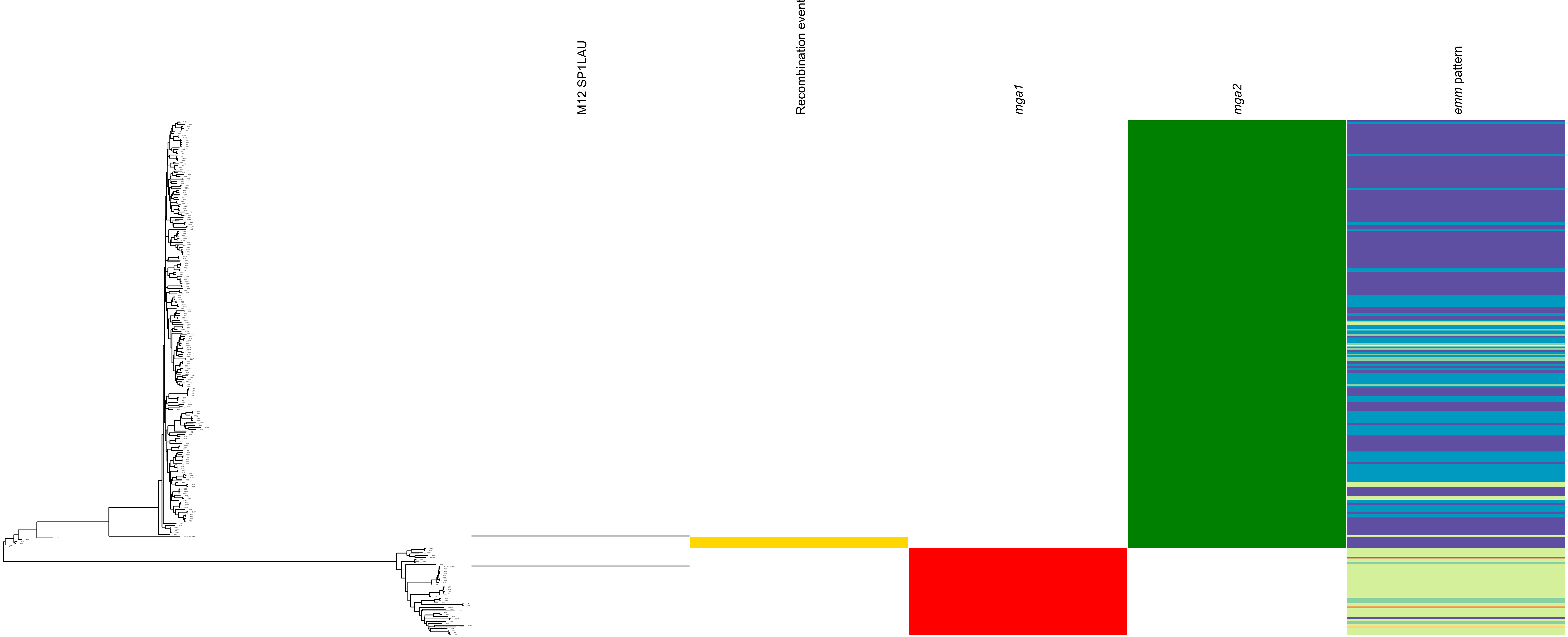

S6b: Phylogram of the *mga-1* and *mga-2* intergenic and coding sequences in 944 genomes. The unique alleles in each of the 125 *emm*-types segregated into three clades corresponding to *mga-1* (red), *mga-2* (green), and a recombination event representing the chimeric combination of *mga-2* downstream of a recombined *mga-1* intergenic region (gold). Silver labels represent the two *mga* loci observed in the *emm12* SP1LAU genome. The evolutionary history was inferred using the Neighbour-Joining method from 1000 replicates (MEGAX). The evolutionary distances were computed using the Maximum Composite Likelihood method and are in the units of the number of base substitutions per site. This analysis involved 289 nucleotide sequences of 2453 polymorphic sites.
