# Supplementary material for: In silico characterisation of stand-alone response regulators of Streptococcus pyogenes
Source: PLoS One. 2020 Oct 19;15(10):e0240834. doi: 10.1371/journal.pone.0240834 (PMC7571705; doi:10.1371/journal.pone.0240834)
Supplement: S4 Fig — Dendrograms of GAS response regulator CDSs (comR, rofA, ralp3, lrp, and copY) and IGR (crgR). (PDF) [file pone.0240834.s008.pdf]

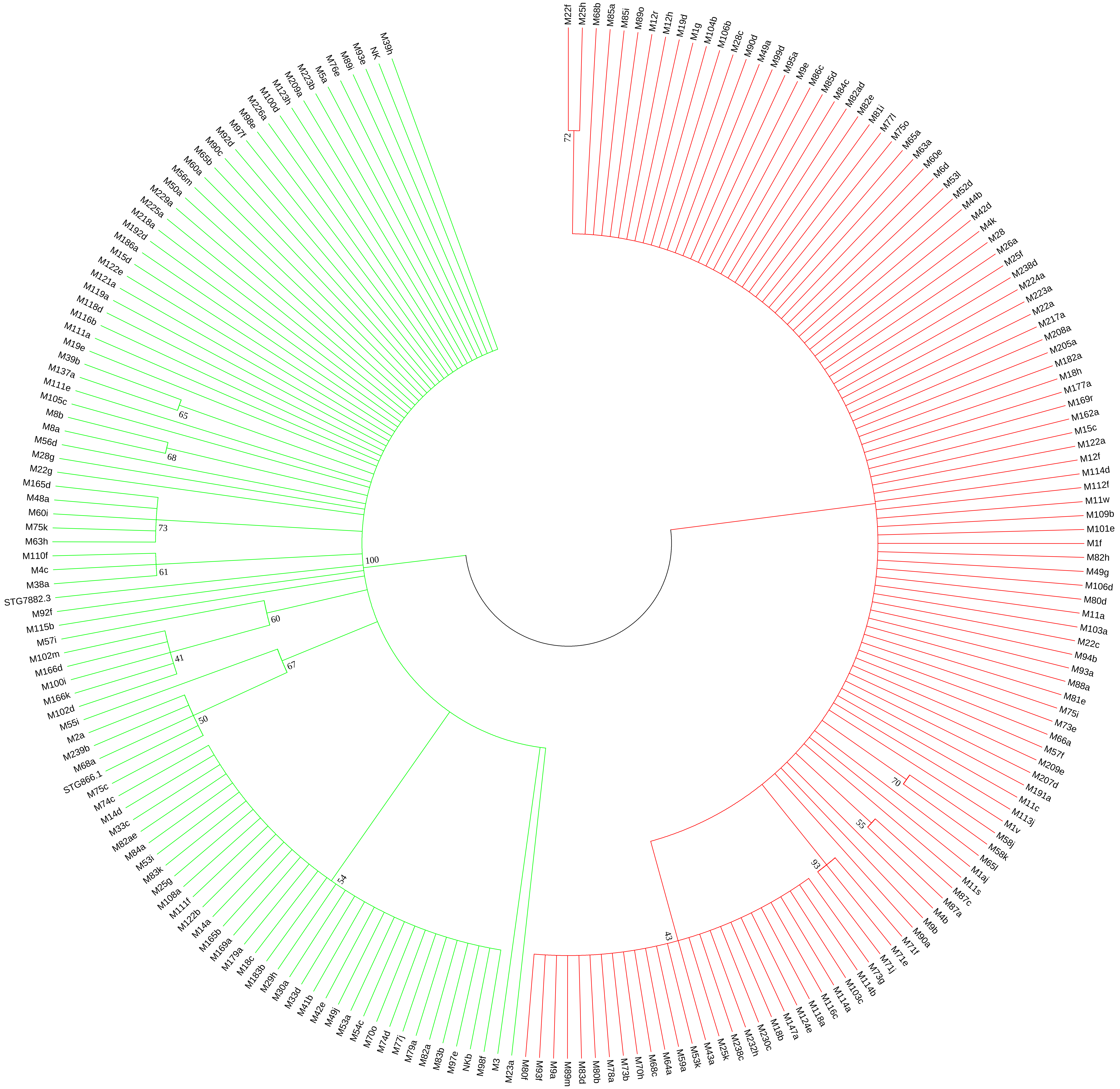

S7 Figure 1: Dendrogram of comR gene coding sequences in 944 genomes. The unique alleles in each of the 125 *emm*-types segregated into two clades corresponding to comR-M1 (red) and comR-M3 (green) (Mashburn-Warren et al 2012). Bootstrap values (percentage from 1000 replicates) of greater than 40% are shown at the bifurcating nodes.

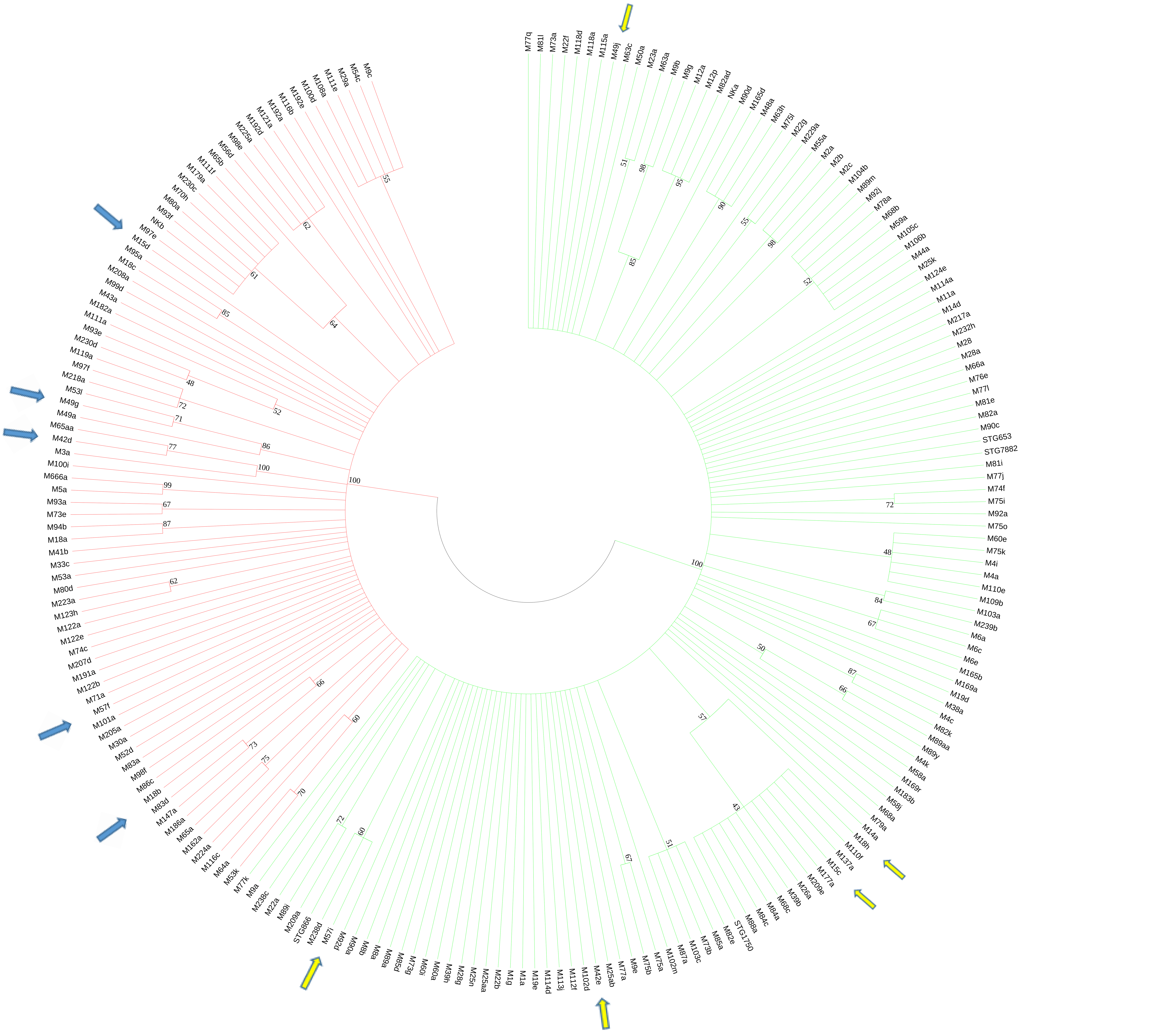

S7 Figure 2: Dendogram of the unique rofA (green) and nra (red) alleles in each of the 125 *emm*-types represented in 944 GAS genomes. The blue and yellow arrows indicate *rofA* and *nra* (n=5) alleles of *emm*-types that encoded multiple FCT-types. Bootstrap values (percentage from 1000 replicates) of greater than 40% are shown at the bifurcating nodes.

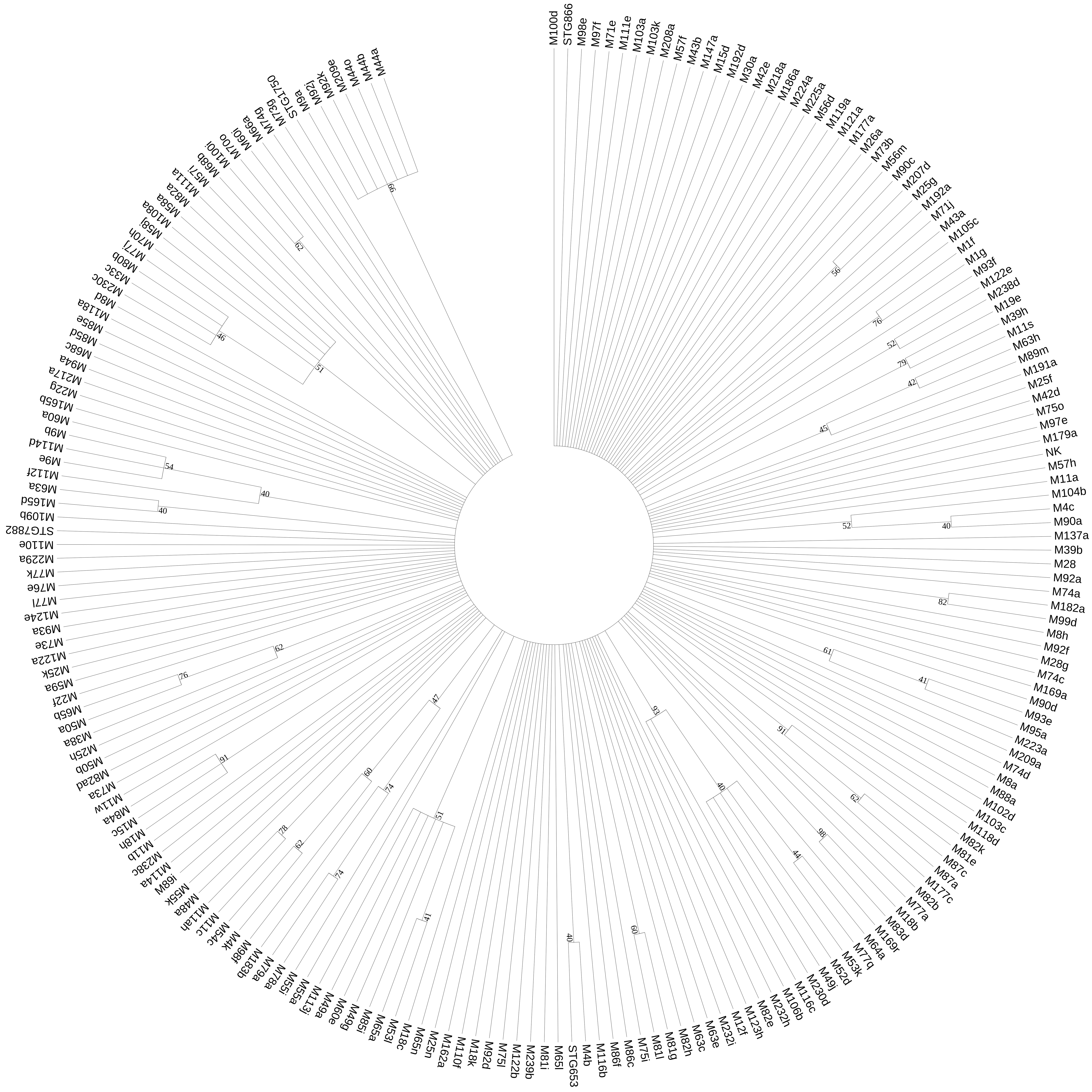

S7 Figure 3: Dendrogram of ralp3 gene coding sequences in 944 genomes. Bootstrap values (percentage from 1000 replicates) of greater than 40% are shown at the bifurcating nodes representing the unique alleles in each of the 111 emm-types.

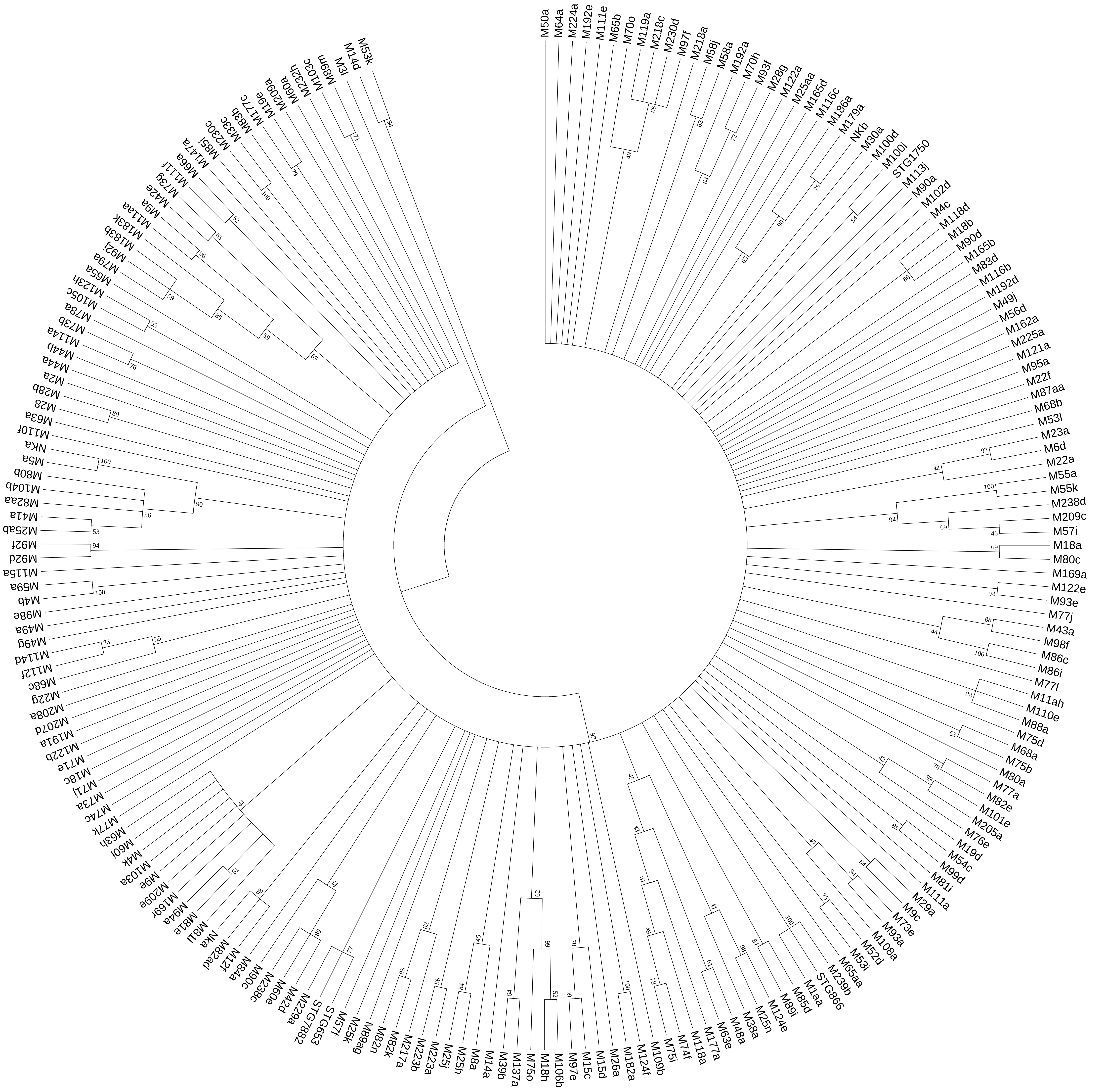

S7 Figure 4: Dendrogram of lrp gene coding sequences in 944 genomes. Bootstrap values (percentage from 1000 replicates) of greater than 40% are shown at the bifurcating nodes representing the unique alleles in each of the 125 emm-types.

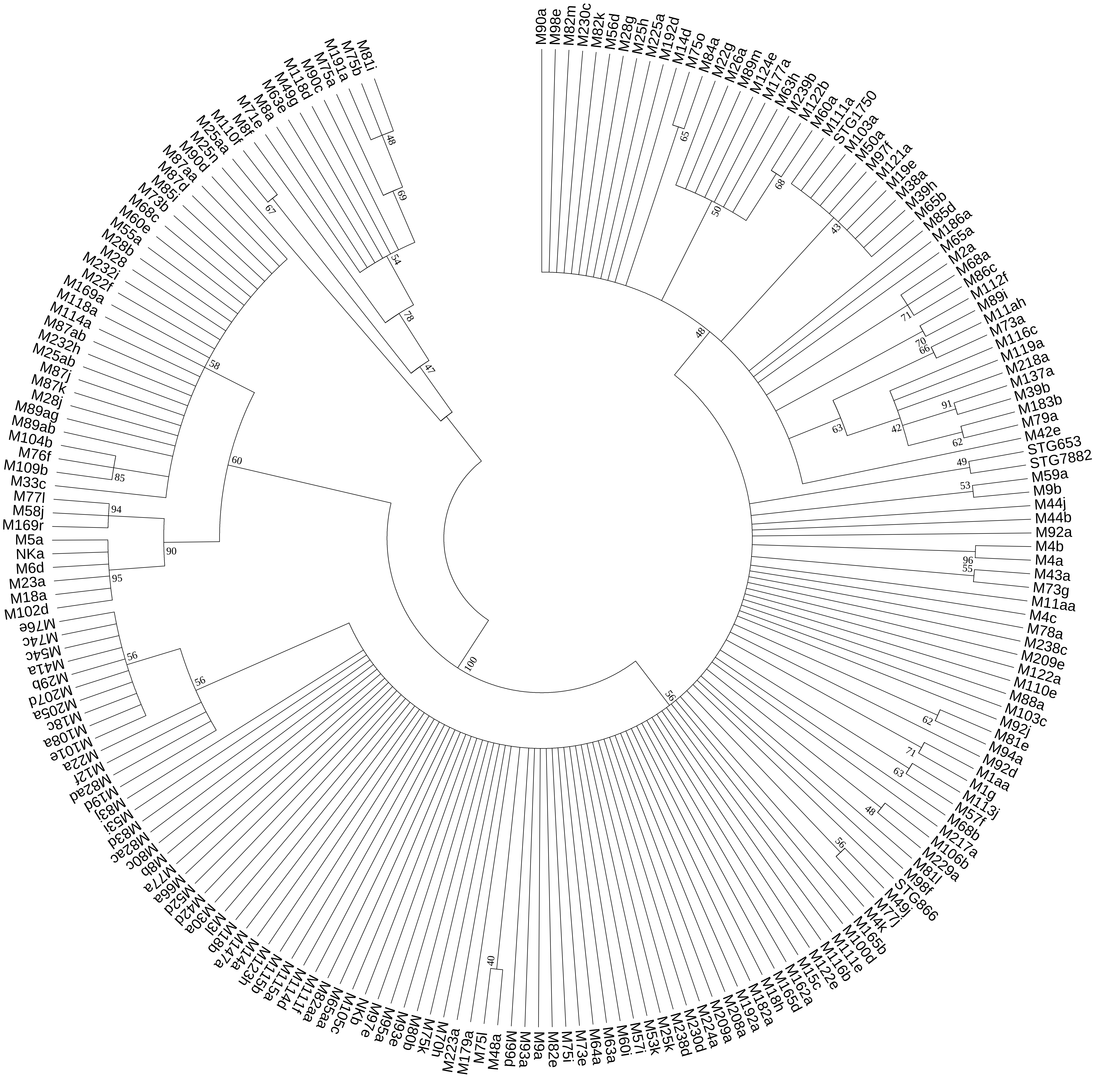

S7 Figure 5: Dendrogram of copY gene coding sequences in 943 genomes. Bootstrap values (percentage from 1000 replicates) of greater than 40% are shown at the bifurcating nodes representing the unique alleles in each of the 125 emm-types.

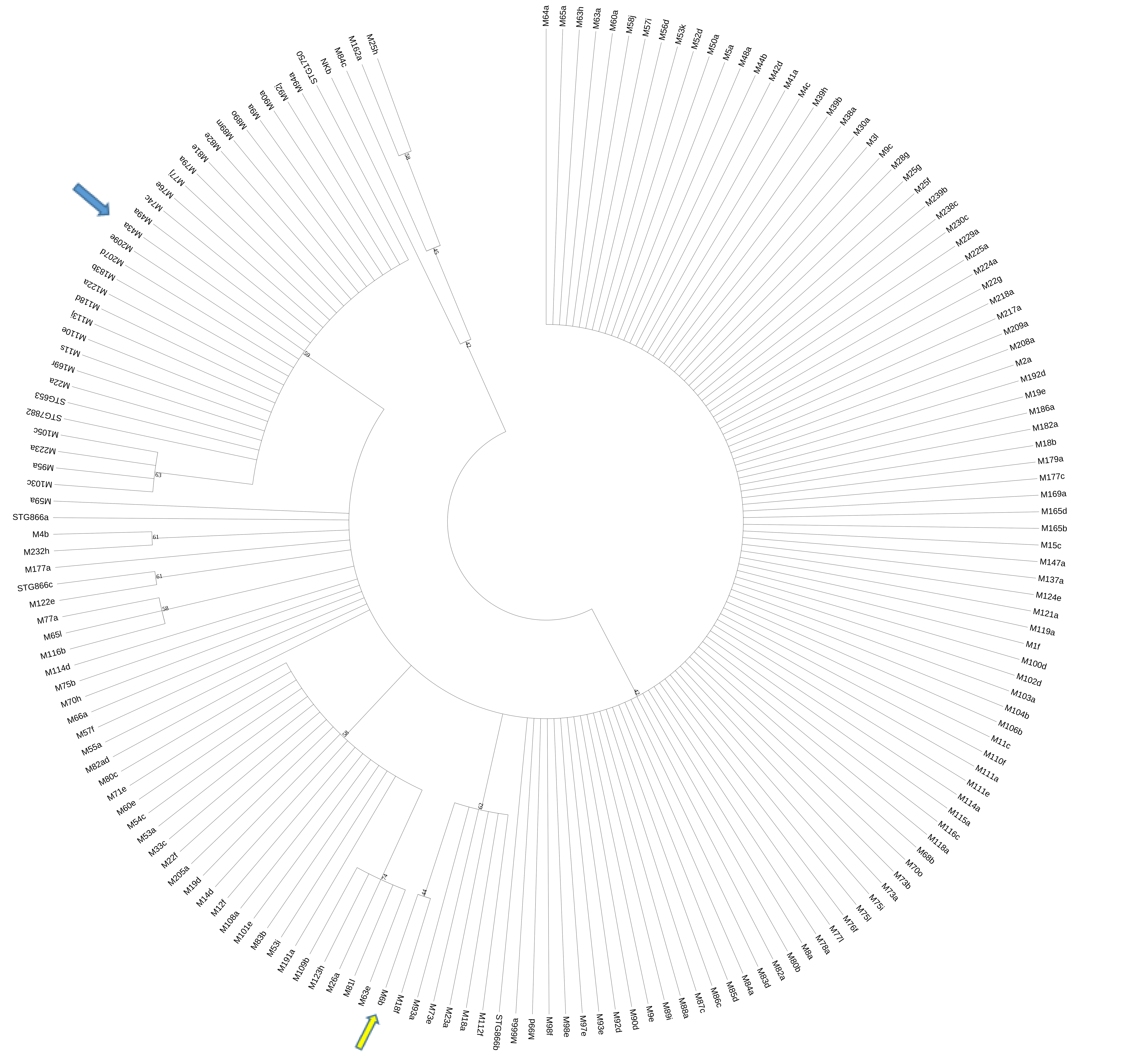

S7 Figure 6: Dendogram of the unique intergenic regions upstream of crgR (IcrgR) in each of the 125 *emm*-types represented in 944 GAS genomes. The blue and yellow arrows indicate the IcrgR alleles of *emm49* NZ131 and *emm6* JRS4 reference genomes, respectively. Bootstrap values (percentage from 1000 replicates) of greater than 40% are shown at the bifurcating nodes.
