# Supplementary material for: In silico characterisation of stand-alone response regulators of Streptococcus pyogenes
Source: PLoS One. 2020 Oct 19;15(10):e0240834. doi: 10.1371/journal.pone.0240834 (PMC7571705; doi:10.1371/journal.pone.0240834)
Supplement: S5 Fig — Neighbour joining phylogenetic tree of 3551 SNPs generated from an alignment of 35 response regulator genes. (PDF) [file pone.0240834.s009.pdf]

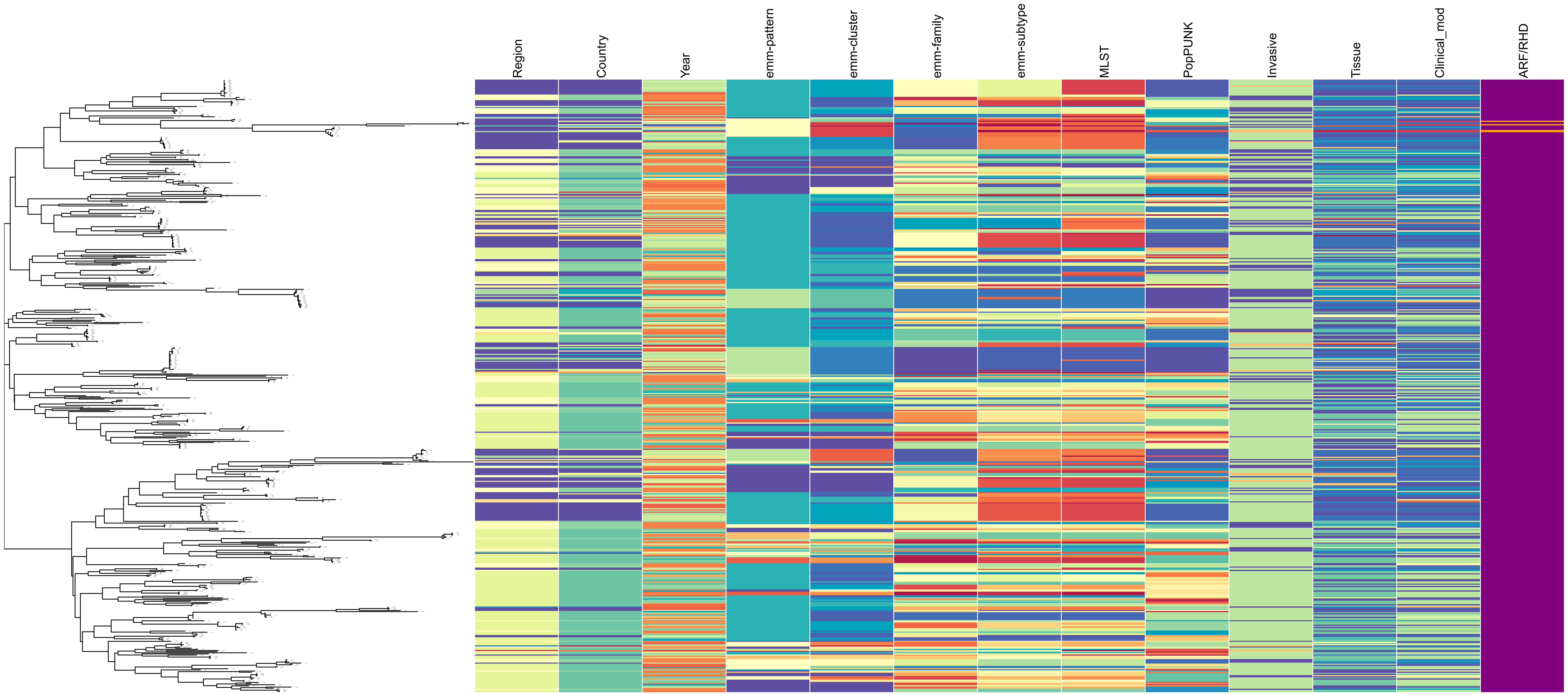


S8: Neighbour joining phylogenetic tree of 3551 SNPs generated from an alignment of 35 response regulator genes.
